# miRNA Polymorphisms and Risk of Cardio-Cerebrovascular Diseases: A Systematic Review and Meta-Analysis

**DOI:** 10.3390/ijms20020293

**Published:** 2019-01-12

**Authors:** Milad Bastami, Jalal Choupani, Zahra Saadatian, Sepideh Zununi Vahed, Yaser Mansoori, Abdolreza Daraei, Hossein Samadi Kafil, Andrea Masotti, Ziba Nariman-saleh-fam

**Affiliations:** 1 Department of Medical Genetics, Faculty of Medicine, Tabriz University of Medical Sciences, Tabriz 5166614766, Iran; mi.bastami@live.com; 2 Immunology Research Center, Tabriz University of Medical Sciences, Tabriz 5166614766, Iran; chupani.genetic@gmail.com; 3 Student Research Committee, Faculty of Medicine, Shahid Beheshti University of Medical Sciences, Tehran 1985717443, Iran; z.saadatian@yahoo.com; 4 Kidney Research Center, Tabriz University of Medical Sciences, Tabriz 5166614756, Iran; sepide.zununi@gmail.com; 5 Noncommunicable Diseases Research Center, Fasa University of Medical Sciences, Fasa 7461686688, Iran; fums.mansoori@gmail.com; 6 Department of Genetics, Faculty of Medicine, Babol University of Medical Sciences, Babol 4617647745, Iran; a.daraei@mubabol.ac.ir; 7 Drug Applied Research Center, Tabriz University of Medical Sciences, Tabriz 5165665811, Iran; kafilhs@tbzmed.ac.ir; 8 Bambino Gesù Children’s Hospital-IRCCS, Research Laboratories, Viale di San Paolo 15, 00146 Rome, Italy; 9 Women’s Reproductive Health Research Center, Tabriz University of Medical Sciences, Tabriz 5138663134, Iran

**Keywords:** microRNA, polymorphism, meta-analysis, cardiovascular disease, cerebrovascular disease, coronary artery disease, ischemic stroke

## Abstract

Recently extensive focus has been concentrated on the role of miRNAs in the initiation and progression of cardio-cerebrovascular diseases (CCDs) which constitute a range of conditions including cardiovascular diseases (CVDs, especially coronary artery disease (CAD)), congenital heart disease (CHD) and cerebrovascular diseases (CBVDs, especially the ischemic stroke (IS)). An increasing number of studies are evaluating the association between different miRNA polymorphisms and risk of CCDs, but results have been inconclusive. This study represents a comprehensive systematic review and meta-analysis of the association between miRNA polymorphisms and risk of CCDs. PubMed, Embase, Scopus, and Web of Science were queried to identify eligible articles. Odds ratios and 95% confidence intervals were used to assess the association of miRNA polymorphisms with CCD susceptibility. A total of 51 eligible articles evaluating the association of 31 miRNA polymorphisms were identified. Meta-analysis was performed for six miRNA polymorphisms. miR-146a rs2910164 (30 studies: 13,186 cases/14,497 controls), miR-149 rs2292832 (Nine studies: 4116 cases/3511 controls), miR-149 rs71428439 (Three studies: 1556 cases/1567 controls), miR-196a2 rs11614913 (20 studies: 10,144 cases/10,433 controls), miR-218 rs11134527 (Three studies: 2,322 cases/2,754 controls) were not associated with overall CCD. miR-499 rs3746444 was associated with CCD (20 studies: 9564 cases/8876 controls). In the subgroups, rs2910164 and rs3746444 were only associated with CVDs, especially CAD. In conclusion, the results support the existence of a role for miR-146a rs2910164 and miR-499 rs3746444 in determining susceptibility to CCDs, especially CAD.

## 1. Introduction

Cardio-cerebrovascular diseases (CCDs) consist of several clinically heterogeneous medical conditions which are among the leading causes of death worldwide. Despite significant recent developments in prevention, diagnosis and clinical management, cardiovascular diseases (CVDs) and cerebrovascular diseases (CBVDs) remain a major challenge imposing a heavy burden on healthcare systems. Among different clinical manifestations of CVD, coronary artery disease (CAD), which is characterized by significant stenosis in a major pericardial artery, is more prevalent and bears the larger burden. CBVD, a range of conditions influencing brain and cerebral arteries, represents a major source of morbidity and mortality [1,2]. A stroke is a clinically important manifestation of CBVD that is presumed to be of vascular origin and is characterized by rapidly developing signs of focal or global disturbance of cerebral functions lasting >24 h or leading to death [1]. In most cases, it is caused by an ischemic stroke (IS) and cerebral infarction.

It has been shown that a range of environmental and genetic factors contribute to the risk of CCDs. Functional alteration of several protein coding genes has been linked to CCDs pathogenesis, but it was not until recently that an extensive focus has been concentrated on the role of noncoding RNAs, especially microRNAs (miRNAs), in the initiation and progression of CCDs. miRNAs are small endogenous regulatory RNAs involved in the post-transcriptional regulation of most protein coding genes. They regulate key cellular functions and most biological processes that are altered during the initiation and progression of CCDs. Studies have demonstrated that single nucleotide polymorphisms (SNPs) residing in miRNA genes may modulate biological functions of these regulatory molecules by altering their processing, expression, and/or targeting efficiency. Consequently, an increasing number of studies have evaluated the association between different miRNA polymorphisms and risk of CCDs, but results are inconclusive. This study represents a comprehensive systematic review and meta-analysis of the association between miRNA polymorphisms and CCDs.

## 2. Results

### 2.1. Study Characteristics

**Figure 1:**
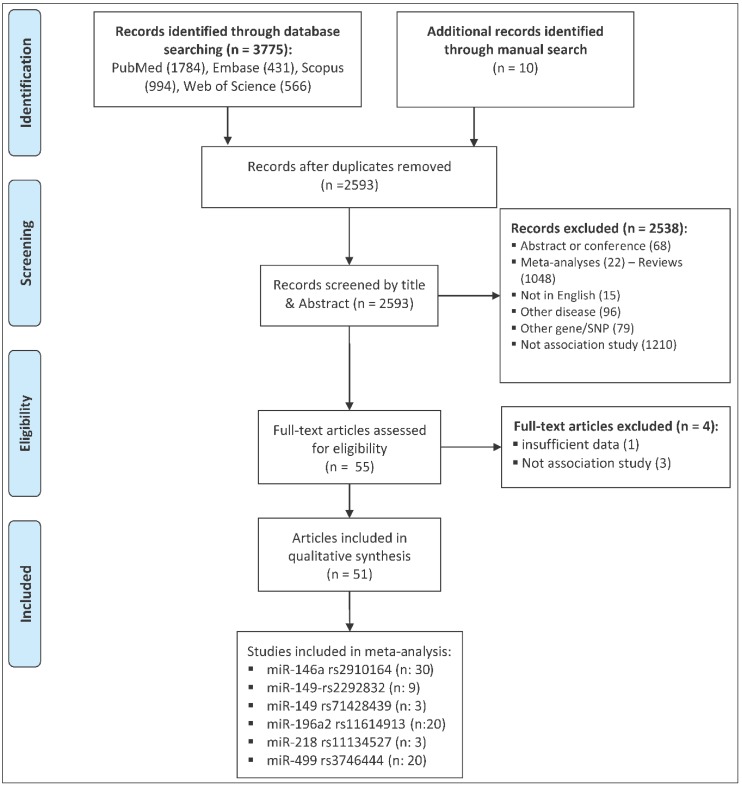
The flow diagram of the study selection process.

The process of selecting eligible studies is depicted in Figure 1. A total of 3785 articles were identified from different sources that are outlined in the materials and methods section and were screened by reading titles and abstracts. A total of 3730 articles were excluded, in which 1192 articles were duplicates, 1210 articles were not association studies, 68 articles were abstracts or conference meetings, 22 articles were systematic review/meta-analysis, 1048 articles were review manuscript, 15 articles were not written in English, 96 articles were related to other diseases, and 79 articles were related to other genes or polymorphisms. The full texts of the remaining 55 articles were evaluated and four additional articles were further excluded. Of these, three articles were excluded because they did not have an association study design. One article was excluded because the study cohort included patients with peripheral artery atherosclerosis as well as patients with CCDs, and authors did not report the genotype counts of CCD patients separately [3]. Finally, a total of 51 eligible articles remained [4,5,6,7,8,9,10,11,12,13,14,15,16,17,18,19,20,21,22,23,24,25,26,27,28,29,30,31,32,33,34,35,36,37,38,39,40,41,42,43,44,45,46,47,48,49,50,51,52,53,54]. A total of 31 miRNA polymorphisms were evaluated in these articles (Table 1 and Table S1). The number of studies per SNP was less than three for 25 out of 31 miRNA polymorphisms, and, therefore, studies related to these miRNAs were not included in the meta-analysis. Table S1 summarizes the main characteristics of studies related to the 25 miRNA polymorphisms that were not included in the meta-analysis. 

The meta-analysis was performed for the remaining six miRNA polymorphisms, including miR-146a rs2910164 (30 studies, 13,186 cases and 14,497 controls), miR-149 rs2292832 (nine studies, 4116 cases and 3511 controls), miR-149 rs71428439 (three studies, 1556 cases and 1567 controls), miR-196a2 rs11614913 (20 studies, 10,144 cases and 10,432 controls), miR-218 rs11134527 (three studies, 2322 cases and 2754 controls) and miR-499 rs3746444 (20 studies, 9564 cases and 8876 controls). Table 1 represents main characteristics of studies included in the meta-analysis. 

### 2.2. The Association of miR-146a rs2910164 and CCD Risk

**Figure 2:**
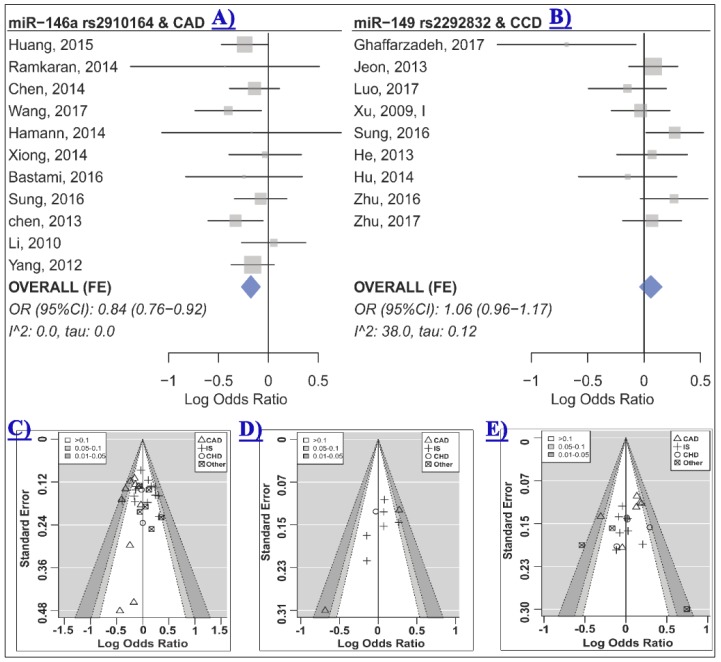
(**A**) The forest plot of association between miR-146a rs2910164 and CAD under the heterozygote model (GC vs. CC) [4,7,8,18,19,24,33,38,39,44,46]; (**B**) the forest plot of association between miR-149 rs2292832 and CCD risk under the heterozygote model (CT vs. TT) [17,22,29,30,34,38,42,45,53]; (**C**)–(**E**) are contour-enhanced funnel plots for meta-analysis of the association between miR-146a rs2910164 (**C**), miR-149 rs2292832 (**D**) or miR-196a2 rs11614913; (**E**) CCD risk under the heterozygote contrast. The contours show various levels of statistical significance of the points/studies (i.e., *p*: 0.1, *p*: 0.05, *p*: 0.01). In particular, the white region in the middle corresponds to *p* values >0.10, the gray-shaded region corresponds to *p* values: 0.10–0.05, the dark gray-shaded region corresponds to *p* values: 0.05–0.01, and the region outside of the funnel corresponds to *p* values <0.01.

Table 2 represents the summary of the meta-analysis results. A meta-analysis of 30 studies, which included 13,186 cases and 14,497 controls, revealed no significant association between miR-146a rs2910164 and risk of cardio-cerebrovascular disease under any genetic model (Table 2 and Figure S1). Subgroup analysis based on the disease category (either CVD or CBVD) and the disease type (either IS or CAD) was also performed, and results showed a significant association between miR-146a rs2910164 and risk of CVD, especially CAD, under the heterozygote and dominant contrasts (Table 3, Figure 2A and Figure S2). No significant association was observed in the CBVD or IS subgroups (Table 3).

The significant association between study heterogeneity that was observed in the overall analysis (Table 2) was present in a number of subgroups (Table 3). Meta-regression showed that the category of disease may significantly contribute to the observed heterogeneity in the homozygote (*R*^2^: 30.7%, test of moderators *p*: 0.02, residual I^2^: 58.0%, τ: 0.24, residual heterogeneity *p* < 0.01), the heterozygote (*R*^2^: 80.2%, test of moderators *p*: 0.008, residual I^2^: 9.7%, τ: 0.05, residual heterogeneity *p*: 0.32), the dominant (*R*^2^: 45.8%, test of moderators *p*: 0.01, residual I^2^: 41.7%, τ: 0.12, residual heterogeneity *p*: 0.01) and the allelic (*R*^2^: 31.6%, test of moderators *p*: 0.02, residual I^2^: 59.6%, τ: 0.11, residual heterogeneity *p* <0.01) contrasts.

The control group in two out of 30 studies deviated from HWE [24,32]. HWD sensitivity analysis showed that excluding these studies influenced the homozygote model of the CVD and the CAD subgroups (Table 3 and Table S2). Therefore, analyses were adjusted for departure from HWE and results were in agreement with the original analyses, indicating that miR-146a rs2910164 was not associated with CVD or CAD under the homozygote model (Table 3, CVD OR_HWD_ (95% CI): 0.84 (0.67–1.06), *p*: 0.13, I^2^: 64.0, τ: 0.26; CAD OR_HWD_ (95% CI): 0.82 (0.65–1.04), *p*: 0.09, I^2^: 64.9, τ: 0.26). No statistical evidence of funnel plot asymmetry was observed (Table 2 all *p* > 0.05, Figure 2C and Figure S3). 

### 2.3. The Association of miR-149 rs2292832 and CCD Risk

Meta-analysis of nine studies which included 4,116 cases and 3,511 controls revealed no evidence of an association between miR-149 rs2292832 and CCD risk under any genetic model (Table 2, Figure 2B and Figure S4). When subgrouped by the disease category or the disease type, a significant association was observed in some subgroups (Table 4). Heterogeneity was not statistically significant in most analyses, and therefore, a fixed-effects model was used in most cases (Table 2 and Table 4). Visual inspection of the funnel plots and contour-enhanced funnel plots revealed no obvious evidence of asymmetry due to publication bias based on statistical significance (Figure 2D). Statistical testing for asymmetry in funnel plots was not performed due to the limited number of studies in the meta-analysis. However, the “trim and fill” method was used to detect possible publication bias and to evaluate the sensitivity of the results (Figure S5). The results suggested one, three, and two missing studies for the homozygote, the heterozygote, and the dominant model, respectively (Figure S5). The filled data yielded free of publication bias ORs consistent with the original analyses and conceded that rs2292832 was not associated with CCD under these models (bias-free OR (95% CI): Homozygote 1.17 (0.88–1.57), heterozygote 1.12 (0.93–1.35), and dominant 1.13 (0.92–1.37)). The trim and filled method suggested that there was no missing study for the recessive and allelic models.

### 2.4. The Association of miR-149 rs71428439 and CCD Risk

Three studies with a total of 1556 cases and 1567 controls were included in the meta-analysis of miR-149 rs71428439 and CCD risk. The meta-analysis showed no evidence of an association between this miRNA polymorphism and risk of CCD (Table 2). Subgroup analysis was not performed for this miRNA polymorphism because of the limited number of studies. Moreover, the number of studies was not sufficient for testing the asymmetry of the funnel plot. As a significant heterogeneity was present in all analyzed models (Table 2), and the random effects model was used in the analyses.

### 2.5. The Association of miR-196a2 rs11614913 and CCD Risk

**Figure 3:**
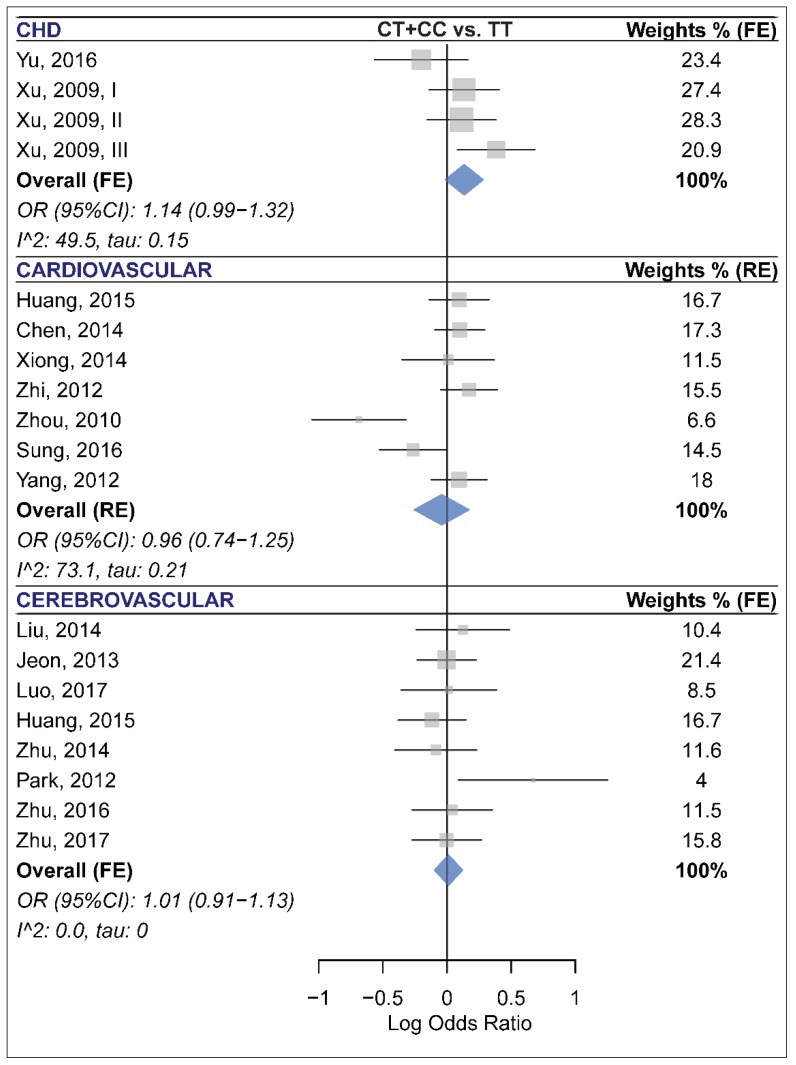
Forest plots of the meta-analysis of the association between miR-196a2 rs11614913 and risk of congenital heart disease (**top** panel) [45,48], cardiovascular disease (**middle** panel) [7,19,38,44,46,50,52], and cerebrovascular disease (**bottom** panel) [20,22,27,29,31,42,53,54].

Meta-analysis of 20 studies with a total of 10,144 cases and 10,433 controls revealed that miR-196a2 rs11614913 was not associated with CCD risk under any of the analyzed genetic models (Table 2 and Figure S6). In subgroup analyses (Table 5), this miRNA polymorphism was not associated with risk of CHD, CVD or CBVD (Figure 3). Moreover, no significant association was observed in the CAD or IS subgroups (Table 5). The statistically significant heterogeneities that were present in the overall analyses, especially the homozygote model (Table 2, τ: 0.23, I^2^: 60.2%), were substantially reduced in most cases when analyses were subgrouped by the disease category or the disease type variable. Specifically, heterogeneity was low in all contrasts of the CAD, IS and CBVD subgroups (Table 5). Moreover, the Galbraith plot analysis identified the study by Zhou et al. [52] as a contributor to the observed between-study heterogeneity in almost all contrasts (Figure S7). After excluding this study, the heterogeneity was substantially reduced in the homozygote model (I^2^ (τ) from 60.2 (0.23) to 37.2 (0.14)), the heterozygote (I^2^ (τ) from 41.2 (0.13) to 23.3 (0.08)), the dominant (I^2^ (τ) from 49.5 (0.14) to 19.6 (0.069)), the recessive (I^2^ (τ) from 60.3 (0.19) to 49.1 (0.15)) and the allelic (I^2^ (τ) from 59.6 (0.11) to 33.4 (0.063)) model. However, excluding this study had no dramatic impact on the pooled estimates and did not alter the results (OR (95% CI) homozygote: 1.06 (0.95–1.20), heterozygote: 1.04 (0.96–1.13), dominant: 1.05 (0.97–1.13), recessive: 1.04 (0.94–1.16), allelic: 1.04 (0.98–1.09)). Furthermore, no visual or statistical evidence of funnel plot asymmetry due to publication bias was observed (All *p* values > 0.05, Table 2, Figure 2E and Figure S8). 

### 2.6. The Association of miR-218 rs11134527 and CCD Risk

Three studies with a total of 2322 cases and 2754 controls were included in the meta-analysis of miR-218 rs11134527 and CCD risk. No significant association between this polymorphism and CCD risk was observed (Table 2). Subgroup analysis and tests of funnel plot asymmetry were not performed for this miRNA polymorphism because of the limited number of studies. 

### 2.7. The Association of miR-499 rs3746444 and CCD Risk

For this polymorphism, a total of 20 studies including 9564 cases and 8876 controls were included in the meta-analysis. The calculation of ORs and 95% CIs under the homozygote and recessive models was not possible for one study [20] because the authors had observed no subject with the GG genotype in their cohort. The random-effects meta-analysis, which is summarized in Table 2 and Figure S9, suggested an association between rs3746444 and CCD under the homozygote (GG vs. AA, OR_RE_ (95% CI): 1.41 (1.06–1.87), *p*: 0.02, τ: 0.42, I^2^: 59.7), the recessive (GG vs. AA+GA, OR_RE_ (95% CI): 1.35 (1.03–1.77), *p*: 0.03, τ: 0.40, I^2^: 57.7) and the allelic (G vs. A, OR_RE_ (95% CI): 1.16 (1.03–1.30), *p*: 0.02, τ: 0.20, I^2^: 71.3) models. The lower 95% CI limit of the pooled estimates under the dominant contrast was close to the null value leading to a non-significant but borderline association under this model (Table 2, GA+GG vs. AA, OR_RE_ (95% CI): 1.15 (0.99–1.32), *p*: 0.05, τ: 0.22, I^2^: 69.0).

**Figure 4:**
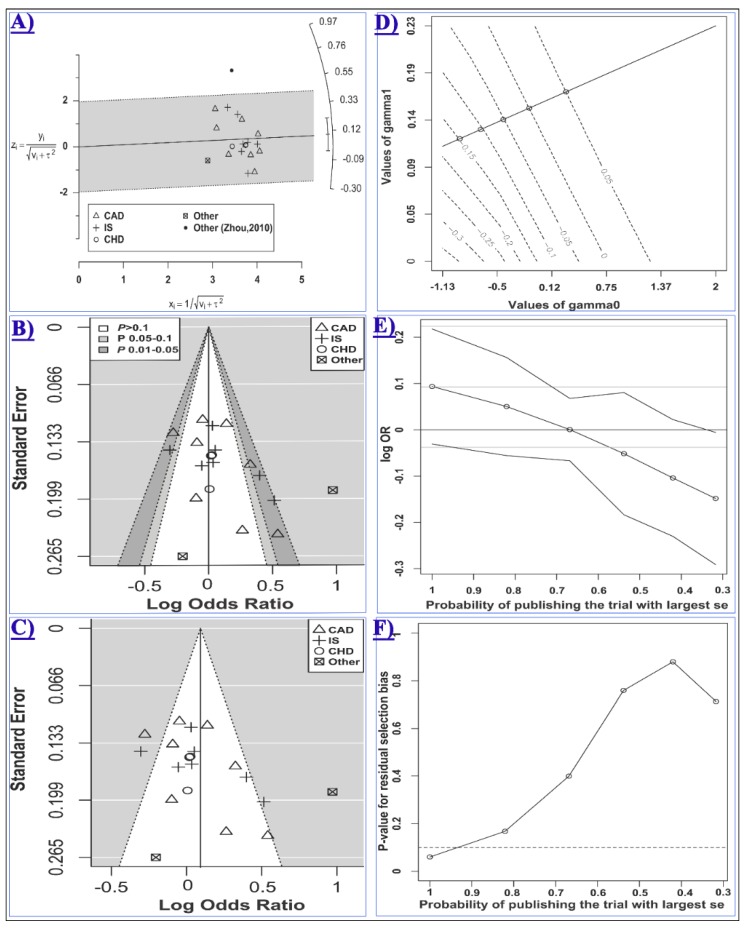
Meta-analysis of the association between miR-499 rs3746444 and CCD risk assuming the heterozygote model (GA vs. AA). In panels A, B and C, studies evaluating CAD, IS, CHD, or other types of CCD are shown with the rectangle, the plus sign, the white circle, or the cube symbol, respectively. (**A**) The Galbraith radial plot shows that the study by Zhou, 2010 [52](the black filled circle) resided outside of the 95% limits, indicating its major contribution to the observed heterogeneity; (**B**) In the contour-enhanced funnel plot, the contours show the various levels of statistical significance of the points/studies (i.e., *p*: 0.1, *p*: 0.05, *p*: 0.01). In particular, the white region in the middle corresponds to *p* values >0.10, the gray-shaded region corresponds to *p* values 0.10–0.05, the dark gray-shaded region corresponds to *p* values 0.05–0.01, and the region outside of the funnel corresponds to *p* values <0.01; (**C**) Is the standard funnel plot for the heterozygote model centered at the random-effects model estimate; (**D**) is the contour plot for the Copas selection model showing the contours of the Copas-estimated effect size as the selection probability varies. The selection probability is a function of γ0 (the horizontal axis) and γ1 (the vertical axis); Plot (**E**) shows how the estimated log OR (95% CI) varies as the probability of publishing the study with the largest standard error decreases; Plot (**F**) shows the *p* value for the residual publication bias at the diminishing probability of publication (i.e., increasing publication bias). The horizontal dashed line represents the *p* value threshold of 0.1. The line crosses the threshold at the point corresponding to the probability of publication greater than 0.9.

Statistically significant heterogeneities were present in the overall analyses, with the most heterogeneous contrast being the recessive model (τ: 0.42, I^2^: 59.7), and, therefore, the random-effects model was used in these analyses. Subgrouping led to a reduction of heterogeneity in some but not all cases (Table 6). Meta-regression showed that at least a part of the heterogeneity may be attributed to the category of disease in the homozygote (*R*^2^: 39.1%, test of moderators *p*: 0.06, residual I^2^: 47.5%, τ: 0.32, residual heterogeneity *p*: 0.01) model. Moreover, the Galbraith plot analysis revealed that the study by Zhou et al. [52] contributed to a considerable portion of the between-study heterogeneity under the dominant and the heterozygote models (Figure 4A). After excluding this study, heterogeneity was substantially reduced in the dominant model (I^2^ (τ) from 69.0 (0.22) to 57.2 (0.16)) and the heterozygote model (I^2^ (τ) from 67.7 (0.22) to 48.3 (0.14)). However, excluding this study had no significant influence on the conclusion of the meta-analysis under these models (dominant OR (95% CI): 1.1 (0.98–1.24), heterozygote OR (95% CI): 1.04 (0.94–1.16)).

**Figure 5:**
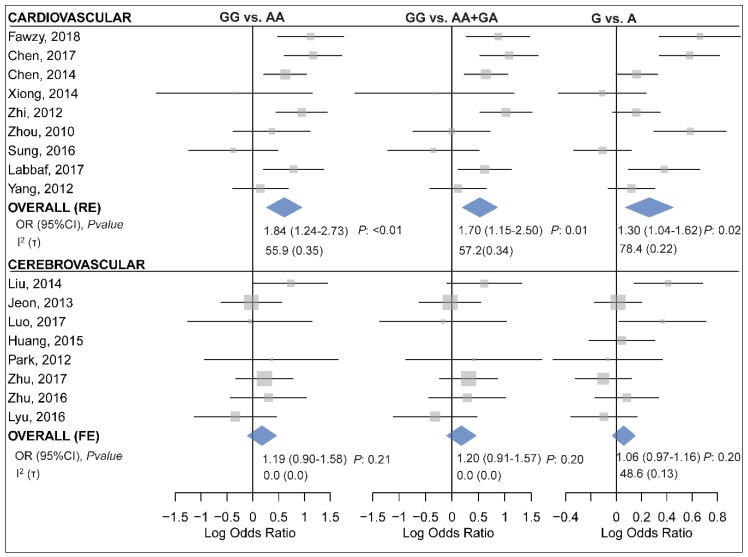
Forest plots for meta-analysis of the association between miR-499 rs3746444 and cardiovascular (**top** panel) [7,10,13,25,38,44,46,50,52] or cerebrovascular diseases (**bottom** panel) [14,20,22,27,29,31,42,53] assuming the homozygote (**left**, GG vs. AA), the recessive (**middle**, GG vs. AA+GA) and the allelic (**right**, G vs. A) model.

When subgrouped by the disease category, statistically significant results were only observed in the CVD subgroup but not in the CHD or CBVD subgroups (Table 6 and Figure 5). Specifically, miR-499 rs3746444 was found to be associated with the risk of CVD under the homozygote (GG vs. AA, OR_RE_ (95% CI): 1.84 (1.24–2.73), *p*: 0.007, τ: 0.35, I^2^: 55.9), the recessive (GG vs. AA+GA, OR_RE_ (95% CI): 1.70 (1.15–2.50), *p*: 0.01, τ: 0.34, I^2^: 57.2) and the allele (G vs. A, OR_RE_ (95% CI): 1.30 (1.04–1.62), *p*: 0.02, τ: 0.22, I^2^: 78.4) contrasts (Table 6). Moreover, the polymorphism was also associated with an increased risk of CAD assuming the homozygote (GG vs. AA, OR_RE_ (95% CI): 1.91 (1.19–3.07), *p*: 0.01, τ: 0.39, I^2^: 62.8) and the recessive (GG vs. AA+GA, OR_RE_ (95% CI): 1.82 (1.19–2.77), *p*: 0.01, τ: 0.34, I^2^: 57.3) models (Table 6). The control group in five out of 20 included studies deviated from HWE [10,20,45,46,52]. Excluding these studies had no significant influence on the overall and subgroup analyses under any genotypic contrast (Table S3). 

Given the presence of substantial heterogeneity in the homozygote and the recessive models (i.e., τ2 > 0.1), the arcsine test of funnel plot asymmetry was used for these contrasts. The Harbord’s test was used for other comparisons. No statistical evidence of asymmetry in the funnel plots was observed (Table 2 and Figure S10). However, as the *p* value of the Harbord’s test for the heterozygote model (i.e., 0.07, Figure 4B,C) was relatively close to the significance threshold, the “trim and fill” and the Copas selection model were used to detect evidence of possible publication bias and explore the sensitivity of results to such a bias. The “trim and fill” method suggested that there was no missing study in the heterozygote model. The Copas selection model suggested modest evidence of publication bias, with the probability of publishing a study with the largest standard error being more than 0.9 and had no significant influence on the overall results (Figure 4D–F and Table 2). The free of publication bias OR (95% CI) under the Copas model was 1.05 (0.94–1.17), which was consistent with the overall analysis.

## 3. Discussion

miRNAs have attracted a great deal of global attention due to their role in regulating the functions of various cell types engaged in the development and pathogenesis of the cardio-cerebrovascular system. Therefore, it is not a surprise that molecular mechanisms governing their regulation can be functionally pertinent to the pathogenesis of CCDs. Among these mechanisms, the influence of polymorphisms on miRNA function and disease susceptibility has remained elusive and controversial irrespective of intensive research, meriting the need for systematic reviews and meta-analyses. This study performed a comprehensive systematic review and identified numerous studies that evaluated the association of miRNA polymorphisms and risk of CCDs. The results indicated that although several miRNA polymorphisms were evaluated for a possible association with risk of CCDs, there was a lack of sufficient data regarding most of these polymorphisms, and only a few polymorphisms were evaluated in more than three studies allowing the meta-analysis to be carried out. Therefore, for a great proportion of the identified miRNA polymorphisms that were assessed, in as few as just one study a definite conclusion cannot be drawn (details of studies that evaluated these polymorphisms are listed in Table S1). In the other side, meta-analyses were carried out for six miRNA polymorphisms with a reasonable number of studies (Table 1).

The following points should be mentioned regarding the methodological aspects of this study. Among several factors leading to the departure of the genotype distributions from HWE, the genotyping error is more relevant to the context of association studies. Although there is no general consensus about the treating of HWE-deviated association studies, it has been recommended that the meta-analysis may benefit from including such studies [55]. However, sensitivity analysis should be performed to assess the possible influences of such studies on the conclusion of the meta-analysis [18,56,57,58]. Therefore, this study included HWE-deviated studies in the meta-analyses while assessing the potential influence of the HWE departure on the overall and subgroup analyses. Visual and statistical inspection of the funnel plot asymmetry has been extensively used to infer the presence of possible publication bias. It is noteworthy that publication bias is only one of the several possible explanations for the observed asymmetry in the funnel plot [59]. The contour-enhanced funnel plot has been shown to facilitate the interpretation of the funnel plot asymmetry [60]. Moreover, it has been shown that between-study heterogeneity may also lead to the funnel plot asymmetry and may influence the results of statistical tests [59]. Therefore, in this study, the contour-enhanced funnel plots were used alongside the standard funnel plots to visually inspect the presence of a possible asymmetry. Moreover, statistical tests for the funnel plot asymmetry were selected and performed in the presence or absence of significant between-study heterogeneity to account for the influence of the heterogeneity on the asymmetry of the funnel plots, as recommended elsewhere [59]. To evaluate the sensitivity of the results to varying degrees of possible publication bias, the “trim and fill” method and Copas selection model were used wherever needed.

### 3.1. The Association of miRNA Polymorphisms with Risk of CCDs

Results of the overall analyses suggested no evidence of an association between miR-146a rs2910164 and risk of CCDs. As indicated by the subgroup analysis (Table 3), this polymorphism may only modulate the risk of CVDs, especially CAD, but not CBVD or IS. According to the results, it is suggested that individuals carrying at least one rs2910164-G allele have a ~15% lower risk of CVD, especially CAD, than those with the CC genotype. This conclusion is also supported by the HWD sensitivity analysis which indicated that the two statistically significant genetics models (i.e., GC vs. CC and GG+GC vs. CC) were not influenced by excluding the study with an HWE-deviated control group. Moreover, the results confirmed that accounting for the departure from HWE may support the results of the original homozygote model. The between-study heterogeneities, which were statistically significant in the overall analyses (Table 2), were reduced in several subgroup comparisons, suggesting the contribution of the disease category/type to the observed heterogeneities. Indeed, meta-regression analysis supported the contribution of the disease category/type to the observed heterogeneity of most genetic models. 

This study found no evidence for the association of miR-149 rs2292832 and risk of CCDs. This polymorphism was associated with the risk of CBVD in the subgroup analysis. In the absence of significant between-study heterogeneity, these results suggest that miR-149 rs2292832 may not contribute to the risk of CCDs assuming any genetic model. However, it should be noted that a relatively limited number of studies evaluated this polymorphism. The number of studies may influence the power of detecting possible publication bias. The funnel plot may not detect publication bias in the presence of a small number of studies. It has been recommended that tests for funnel plot asymmetry should not be performed when there are less than 10 studies in the analyses. Moreover, asymmetry of the funnel plot does not necessarily reflect publication bias and may be caused by other factors. Therefore, tests for evaluating the asymmetry of the funnel plots were not performed for this polymorphism. Instead, the “trim and fill” model was used to evaluate the sensitivity of the results to possible publication bias, and the results confirmed the original findings. More association studies are needed to be able to strongly rule out the possibility of publication bias and to draw a more definite conclusion. This study also found no association between miR-149 rs71428439 or miR-218 rs11134527 and risk of CCDs. It should be noted that the limited number of studies impede our ability to draw a definite inference. Therefore, more association studies for each disease type are necessary to reach a more robust conclusion.

Through pooling the results of 20 studies with a total of 10,144 cases and 10,433 controls, this study revealed that miR-196a2 rs11614913 was not associated with risk of CCDs. The statistically significant between-study heterogeneities, which were observed in the overall analysis (see Table 2), were considerably reduced in most cases when subgrouped by the disease category/type, suggesting a role for this moderator in the observed heterogeneity. Meta-analysis showed evidence for the association of miR-499 rs3746444 with risk of CCD under three genetic models. Results suggest that subjects carrying the GG genotype have a higher risk of CCD compared to individuals with the AA genotype or to those carrying at least one A allele. However, as indicated by the subgroup analysis (Table 5), miR-499 rs3746444 only modifies the risk of CVDs, especially CAD, but not CBVD or IS. Therefore, results suggest that subjects carrying the GG genotype have a higher risk of CVDs, especially CAD, compared to individuals with the AA genotype or to those carrying at least one A allele. This conclusion is further supported by the HWD sensitivity analysis indicating that excluding HWE-deviated studies does not influence the conclusion of the meta-analysis (summarized in Table S3). The disease category/type explained a part of the between-study heterogeneity in the homozygote model. However, the residual heterogeneity was present even after accounting for the effect of the disease category/type moderator, suggesting that other unknown study level moderators may contribute to the heterogeneity. The study by Zhou [52] also partly contributed to the heterogeneity but did not influence the results. Statistical tests for the funnel plot asymmetry were selected and performed in light of the presence or absence of significant between-study heterogeneity to account for the influence of the heterogeneity on the asymmetry of the funnel plots. The results of these tests together with the “trim and fill” method and the Copas selection model suggested no strong evidence for publication bias and confirmed that the results are not sensitive to possible publication bias.

### 3.2. Possible Pathogenetic Mechanisms and Effects of miR-146a rs2910164

It has been shown that polymorphisms residing in pre-miRNAs may contribute to complex diseases through a number of possible mechanisms. miRNA polymorphisms may alter the secondary structure of pre-miRNA and influence its processing and maturation, manifesting as an altered mature miRNA expression or a modified miRNA-5p/3p ratio [61]. Alternatively, polymorphisms inside mature miRNAs may modify the repertoire of genes targeted by the corresponding miRNA through either attenuating/destroying existing miRNA-mRNA hybrids or creating novel interactions [61]. Although polymorphisms in the critical seed region (i.e., nucleotide 2–8 from the 5′ end of mature miRNA) is usually considered to confer more impacts, other nucleotides in the mature miRNA but outside of the seed region may also influence, though less intensely, the repertoire or the strength of the interactions. Rs2910164 is a C>G substitution which induces a mispairing in the 3′ arm of the hsa-miR-146a precursor (MI0000477) and affects the third base in the seed region of hsa-miR-146a-3p (MIMAT0004608). Increasing evidence suggests that rs2910164 influences the processing and expression of miR-146a. However, there is no consensus regarding the alterations that are induced by either allele of this polymorphism. A number of studies have reported that the C allele is correlated with increased expression of the lead miRNA as compared to the G allele. For example, two studies have shown that CAD patients carrying the CC genotype had higher levels of miR-146a in their peripheral blood mononuclear cells (PBMCs) compared to those with the GG genotype [33,44]. A gradual increase in PBMC miR-146a level with the addition of each C allele has been reported in CAD patients [44].

In contradiction, there are also reports indicating that the G allele may induce increased miR-146a expression in complex diseases ranging from type 2 diabetes mellitus to thyroid carcinoma [62,63,64]. This discrepancy raises the possibility that some disease-specific and/or cell type/tissue-specific factors may modify the effect of polymorphisms on miRNA function. As a matter of fact, in a study on PBMCs of CAD patients and healthy subjects, the correlation between rs2910164 genotypes and miR-146a expression was observed in patients but not in the healthy controls [33]. A similar discrepancy between pathological and normal samples in terms of miR-146a genotype-expression correlation was also observed in a study on lung cancer [64]. The present study concluded that the C allele may predispose individuals to CVDs, especially CAD. This observation, along with previous studies demonstrating the correlation of the C allele with miR-146a-5p expression, suggests that the C allele induces the expression of miR-146a-5p in PBMCs, potentially contributing to the observed inflammatory profile of these cells. As an indispensable component of the immune system, PBMCs actively cross-talk with pathophysiological conditions induced by endothelial cell injury, which reflects as a sustained inflammatory profile. Moreover, miR-146a is a well-known modulator of inflammation which inhibits nuclear factor Kappa B (NF-κB) via targeting interleukin-1 receptor-associated kinase 1 (IRAK-1) and tumor necrosis factor receptor-associated factor 6 (TRAF-6) in the TLR pathway. Interestingly, it has been suggested that the rs2910164-CC genotype is correlated not only with increased PBMC expression of miR-146a in CAD patients but also with decreased levels of IRAK1, TRAF6, and NF-κB [33]. Apart from altering expression, rs2910164 resides in the seed region of miR-146a-3p and may potentially influence the repertoire of its target gene. In our previous *in-silico* analyses [4], the potential influence of rs2919164 on miR-146a-3p targeting was investigated by performing a functional annotation clustering analysis on genes that harbor a predicted miR-146a-3p binding either disrupted or created by rs2910164. Results of these analyses suggested that rs2910164 may create novel miR-146a-3p binding sites in 3′-UTR of several genes that are enriched in CAD-related biological processes such as the activation of immune response (GO:0002253), regulation of apoptosis (GO:0042981), and the T-cell receptor signaling pathway (GO:0050852). Furthermore, pre-existing miR-146a-3p binding sites in several genes engaged in inflammatory processes such as cell activation during the immune response (GO:0002263) and mast cell-mediated immunity (GO:0002448) may be disrupted by rs2910164 [4].

### 3.3. Possible Pathogenetic Mechanisms and Effects of miR-499 rs3746444

miR-499 is preferably expressed in cardiac cells and skeletal muscles and plays key roles in heart development. Studies have shown that miR-499 is dynamically-regulated during the differentiation of cardiomyocytes [65,66]. One study on transgenic mice has detected an altered cardiac gene expression following the upregulation of miR-499 and suggested that elevation of miR-499 may predispose mice to cardiac stress-induced dysfunction in a dose-dependent manner [67]. Consistent with its role in modulating cardiac response to stress, it has been shown that miR-499 regulates immediate early gene response [67]. Interestingly, elevated levels of circulating miR-499 following myocardial infarction in rats or human subjects have been reported [68,69]. Furthermore, miR-499 protects rat cardiomyocytes from H_2_O_2_-induced apoptosis via inhibiting the mitochondrial apoptosis pathway which suggests a protective role for miR-499 against H_2_O_2_-induced cardiomyocytes injuries [70]. In endothelial cells, however, miR-499 regulates the inflammatory damage during CAD by targeting PDCD4 through the NF-κβ/TNF-α pathway [71]. Therefore, down-regulation of miR-499 may protect endothelial cells from inflammatory damages during CAD [71]. 

The locus for human miRNA-499 is located in an intronic region of the cardiac β-myosin heavy chain 7B gene (*MYH7B*) [72]. This region has a special structure as different pre-miRNAs are encoded from opposite strands of the same locus. Hsa-miR-499a (miRBase MI0003183, HGNC: MIR499A, GRCh38 chr20: 34990376–34990497 [+]), which is a member of miR-499 precursor family (MIPF0000173), is encoded from the forward strand and processed into two mature miRNAs: hsa-miR-499a-5p (synonym: miR-499) and hsa-miR-499a-3p. Hsa-miR-499b (miRBase MI0017396, HGNC: MIR499B, GRCh38 chr20: 34990400–34990472 [−]) is another miRNA precursor which is encoded from the opposite strand of the same region and processed into hsa-miR-499b-5p and hsa-miR-499b-3p. During recent years, much attention has been paid to functions of mature miRNAs processed from hsa-miR-499a and, currently, there is a lack of data regarding possible functions of hsa-miR-499b. Traditionally, only one of the two mature miRNAs generated from the precursor is considered to be functional, which is most commonly the miRNA processed from the 5p arm, and its complementary species is usually considered to be degraded. However, increasing evidence suggests the co-existence of both 5p and 3p mature miRNAs, albeit in different concentrations [73,74,75].

In some cases, miRNA-5p and -3p excised from the same precursor may target distinct transcripts [76]. Co-expression and regulation of paired miRNAs 5p/3p are under active research. A tissue-dependent regulatory role for the 5p and 3p strands has been reported, indicating that while strand selection may occur in some tissues, both strands may be co-accumulated as miRNA pairs in other tissues [75]. While many studies have focused on the functions of miR-499a-5p (previously known as miR-499 or miR-499-5p), a recent report has shed light on the role of miR-499a-3p in atherosclerosis [77]. This study demonstrated an elevated serum level of miR-499a-3p in CAD patients compared to healthy controls and confirmed that miR-499a-3p promotes proliferation and migration of endothelial cells and vascular smooth muscle cells via directly targeting myocyte enhancer factor 2C (*MEF2C*) at 3′-UTR [77]. These observations merit the need for more experiments to clarify the regulation and co-expression of miR-499a-5p/3p and determine whether miR-499b-5p/3p has atherosclerosis-related functions.

Rs3746444 (GRCh38 chr20:34990448) overlaps both precursors, residing in the seed region of miR-499a-3p and the 3′ portion of miR-499b-5p. Although this polymorphism may potentially influence the structure and function of both miRNA precursors, currently there is insufficient data regarding its influence on miR-499b. It has been shown that the G allele can be correlated with lower miR-499a-5p expression in breast or lung tissues [55,78]. Theoretically, rs3746444 may increase the risk of CCD by either altering miR-499a-5p/3p (or possibly miR-499b-5p/3p) expression or influencing the repertoire of target genes of miR-499a-3p (or possibly miR-499b-5p), or a combination of both scenarios. A recent study provided support for the first scenario [56], demonstrating that rs3746444 can modulate the expression of miR-499a. This study showed that the rs3746444-A allele may be correlated with higher serum levels of miR-499a-5p and lower HDL in a recessive manner (i.e., AA vs. AG+GG). It is not yet clear how, and to what extent, rs3746444 may interfere with miR-499-3p targeting and what implications these changes would have for the pathogenesis of CCDs. Further research is necessary to elucidate polymorphism-induced changes in complex diseases. However, the multicellular nature of CAD and the cell/tissue-specific regulation of miRNAs may potentially complicate this process.

## 4. Materials and Methods

### 4.1. Publication Search

To identify all potentially eligible publications, Embase, PubMed, Web of Science, and Scopus databases were searched using following keywords with respect to specific search tips of each database. (“cardiovascular disease” OR “myocardial infarction” OR “cardiac arrest” OR “heart failure” OR “heart attack” OR “ischemic stroke” OR “ischemic heart disease” OR “ischaemic heart disease” OR “coronary artery disease” OR “coronary heart disease” OR “coronary syndrome” OR “coronary stenosis” OR “congenital heart disease” OR “dilated cardiomyopathy” OR “cardiomyopathy” OR “cardio-cerebrovascular diseases” OR “cerebrovascular diseases” OR “atrial fibrillation” OR “silent brain infarction” OR “lacunar infarction” OR atherosclerosis OR cardiac OR CAD OR CHD OR MI OR CVD) AND (miRNA OR microRNA OR pre-miR OR miR) AND (“single nucleotide polymorphism” OR SNP OR variant OR variation OR polymorphism OR mutation OR locus).

The last search was performed on 1 July 2018. References of review articles, meta-analyses, and other relevant articles were also screened to identify all potentially eligible articles. This meta-analysis was carried out in accordance with the Preferred Reporting Items for Systematic Reviews and Meta-Analyses (PRISMA) statement [57].

### 4.2. Inclusion and Exclusion Criteria

Studies should meet the following criteria to be included: (1) Evaluation of genetic association between miRNA polymorphism and susceptibility to any cardio-cerebrovascular disease with a case-control design. The following ICD-10-CM codes were used: Ischemic heart diseases (I20–I25), other forms of heart disease (I30–I52 and Q20–Q26), cerebrovascular diseases (I60–I69); (2) availability of sufficient data for estimating odds ratio (OR) and its 95% confidence interval (95% CI). A minimum of three studies for each miRNA polymorphism should be met to include the polymorphism in the meta-analysis. Studies that met the following criteria were excluded: (1) Meta-analyses, review articles or abstracts; (2) duplicate publications; (3) studies on animals or cell-lines; (4) studies without a case-control design (5) studies that did not report genotype frequencies.

### 4.3. Data Extraction

Data were extracted from each eligible study and manually checked. Then, items were recorded for each eligible study: The first author, publication year, country, ethnicity, disease category, disease type, source of controls, miRNA name, polymorphism ID, genotyping method, genotype counts for each SNP and number of cases and controls recruited. The disease category was assigned to each study as cardiovascular (CVD), cerebrovascular (CBVD) or congenital heart disease (CHD) and the disease type was considered as either IS (ischemic stroke), CAD (coronary artery disease) or other diseases. 

### 4.4. Statistical Analysis

The Meta package for R was used to perform the meta-analysis [58]. The association of miRNA polymorphisms with cancer was estimated by calculating pooled ORs and their 95% CIs assuming homozygote, heterozygote, dominant, recessive and allelic models. Heterogeneity was assessed using the Chi-squared based Q test [79]. In the presence of significant heterogeneity (i.e., the *p* value of the Q test <0.05 or I^2^ > 50%), the random-effects (RE) model [80] was used to calculate pooled ORs and 95% CIs. Otherwise, the fixed-effects (FE) model was used [81]. The significance of the pooled OR was determined using the Z test (*p* < 0.05 was considered significant). A univariate meta-regression was carried out to identify potential sources of heterogeneity. The Galbraith plot analysis was used to examine the heterogeneity [82]. Subgroup analyses based on the disease category and disease type were performed. The recommendations of other investigators were followed for examining and interpreting the asymmetry of funnel plots [59]. A statistical test for funnel plot asymmetry was not performed when there were fewer than ten studies in the meta-analysis because of low test power to distinguish chance from real asymmetry [59].

In the absence of substantial heterogeneity (i.e., when the estimated heterogeneity variance of log odds ratios, τ2, <0.1), a weighted linear regression test utilizing efficient score and score variance proposed by Harbord et al. [83] was used to detect asymmetry in funnel plots, as the test avoids the mathematical association between the log odds ratio and its standard error while retaining statistical power. Given that false positive results may occur in the presence of high between-study heterogeneity [59], the arcsine test proposed by Rücker et al. [84] which is based on the arcsine transformation of observed risks and explicitly models between-study heterogeneity was used in such cases (i.e., when τ2 > 0.1). Results of asymmetry tests were interpreted in the context of visual inspection of funnel plots. In the presence of evidence for publication bias, the Copas selection model [85,86] or the “trim and fill” approach [87,88] was used to explore the sensitivity of meta-analysis conclusions and adjust for selection bias or funnel plot asymmetry. The Copas selection model was utilized as a way to explore sensitivity of the results to varying levels of possible selection bias, as recommended by empirical evaluations [89,90]. In the present meta-analysis, the following approach with regards to HWE-deviated studies was followed: The departure of genotype distributions from HWE (i.e., HWD) in the control group of each study was evaluated using the Chi-squared or the exact goodness of fit test. Meta-analyses, including the overall and subgroup analyses, were performed considering all eligible studies, including HWD studies. However, to evaluate the possible impacts of HWE-deviated studies, the HWD sensitivity analysis was performed by evaluating the influence of excluding these studies on point estimates and identifying the influenced genotype contrasts. In cases in which excluding HWD studies altered the result of the meta-analysis, the ORs of such studies were adjusted for HWE deviation by means of incorporating the HWE-expected genotype counts in the control group, as recommended [91,92,93,94,95], and the HWD-adjusted pooled ORs were calculated in genotype contrasts. All *p* values were two-sided, and a *p* value < 0.05 was considered statistically significant. All statistical analyses were performed in R (version 3.3.1).

## Figures and Tables

**Table 1:** Main characteristics of studies included in the meta-analysis.

Author	miRNA Polymorphism	Region	Genotyping	Source ^a^	Cases ^b^	Controls ^b^	Condition	HWE ^c^
Bastami, 2016 [4]	miR-146a-rs2910164	Iran	TaqMan	HB	34/155/111	22/128/150	CAD	0.52
Chen, 2013 [8]	miR-146a-rs2910164	China	TaqMan	NA	172/305/181	134/330/194	CAD	0.81
Chen, 2014 [7]	miR-146a-rs2910164	China	PCR-LDR	NA	187/463/269	153/435/301	CAD	0.89
Hamann, 2014 [18]	miR-146a-rs2910164	Germany	HRM	PB	12/74/120	10/73/117	CAD	0.87
Hu, 2014 [30]	miR-146a-rs2910164	China	PCR-RFLP	NA	75/87/34	97/82/26	IS	0.24
Huang, 2015-a [19]	miR-146a-rs2910164	China	TaqMan	HB	266/308/143	237/348/132	CAD	0.87
Huang, 2015-b [20]	miR-146a-rs2910164	China	TaqMan	HB	189/261/81	219/257/55	IS	0.12
Jeon, 2013 [22]	miR-146a-rs2910164	South Korea	PCR-RFLP	HB	360/506/185	211/266/76	IS	0.64
Li, 2010 [24]	miR-146a-rs2910164	China	PCR-RFLP	HB	149/184/82	345/455/210	CVD	0.01
Li, 2014 [26]	miR-146a-rs2910164	China	SNaPshot	HB	73/85/15	111/136/51	LI	0.45
Liu, 2014 [27]	miR-146a-rs2910164	China	PCR-RFLP	HB	85/159/52	116/198/77	IS	0.71
Luo, 2017 [29]	miR-146a-rs2910164	China	SNaPshot	HB	129/130/39	119/139/45	IS	0.74
Lyu, 2016 [14]	miR-146a-rs2910164	China	TaqMan	HB	119/198/61	153/187/38	IS	0.10
Park, 2012 [31]	miR-146a-rs2910164	South Korea	PCR-RFLP	HB	38/56/13	91/113/36	MD	0.97
Qu, 2016 [32]	miR-146a-rs2910164	China	PCR-LDR	NA	355/618/166	483/869/233	IS	0.00
Ramkaran, 2014 [33]	miR-146a-rs2910164	South Africa	PCR-RFLP	NA	13/43/50	9/46/45	CAD	0.69
Shen, 2015 [35]	miR-146a-rs2910164	China	TaqMan	HB	217/283/96	153/177/49	CA	0.91
Sima, 2015 [37]	miR-146a-rs2910164	China	PCR-RFLP	HB	37/100/27	134/254/90	IA	0.13
Sun, 2011 [21]	miR-146a-rs2910164	China	PCR-RFLP	HB	146/170/65	228/304/118	IS	0.38
Sung, 2016 [38]	miR-146a-rs2910164	South Korea	PCR-RFLP	HB	203/242/77	202/260/73	CAD	0.50
Wang, 2013 [40]	miR-146a-rs2910164	China	PCR-RFLP	HB	84/121/45	48/69/17	CHD	0.38
Wang, 2017 [39]	miR-146a-rs2910164	China	MassARRAY	HB	136/155/62	105/179/84	CAD	0.70
Xiong, 2014 [44]	miR-146a-rs2910164	China	PCR-RFLP	HB	113/141/41	97/125/61	CAD	0.10
Xu, 2009, I [45]	miR-146a-rs2910164	China	PCR-RFLP	HB	161/245/95	164/255/86	CHD	0.48
Yang, 2012 [46]	miR-146a-rs2910164	China	TaqMan	NA	272/392/165	271/457/189	CAD	0.92
Zhong, 2016 [51]	miR-146a-rs2910164	China	CE	HB	141/128/28	113/152/35	IS	0.16
Zhou, 2010 [52]	miR-146a-rs2910164	China	PCR-RFLP	HB	78/113/30	120/165/36	DM	0.08
Zhu, 2014 [54]	miR-146a-rs2910164	China	PCR-LDR	HB	145/173/50	132/185/64	IS	0.97
Zhu, 2017 [53]	miR-146a-rs2910164	China	TaqMan	HB	170/267/86	204/251/55	IS	0.58
Zhu, 2016 [42]	miR-146a-rs2910164	China	PCR-RFLP	HB	131/194/71	154/179/45	IS	0.10
Ghaffarzadeh, 2017 [17]	miR-149-rs2292832	Iran	PCR-RFLP	HB	53/124/95	17/79/53	CAD	0.16
He, 2013 [34]	miR-149-rs2292832	China	PCR-RFLP	NA	138/162/57	160/175/38	IS	0.37
Hu, 2014 [30]	miR-149-rs2292832	China	PCR-RFLP	NA	79/76/41	80/89/36	IS	0.24
Jeon, 2013 [22]	miR-149-rs2292832	South Korea	PCR-RFLP	HB	479/472/100	262/238/53	IS	0.97
Luo, 2017 [29]	miR-149-rs2292832	China	SNaPshot	HB	131/127/40	121/136/46	IS	0.50
Sung, 2016 [38]	miR-149-rs2292832	South Korea	PCR-RFLP	HB	227/248/47	263/219/53	CAD	0.51
Xu, 2009, I [45]	miR-149-rs2292832	China	PCR-RFLP	HB	224/233/44	220/236/49	CHD	0.24
Zhu, 2017 [53]	miR-149-rs2292832	China	TaqMan	HB	232/221/70	240/213/57	IS	0.79
Zhu, 2016 [42]	miR-149-rs2292832	China	PCR-RFLP	HB	165/179/52	190/158/30	IS	0.39
Chen, 2014 [7]	miR-149-rs71428439	China	PCR-LDR	NA	375/389/155	384/381/124	CAD	0.07
Chen, 2015 [9]	miR-149-rs71428439	China	Sequencing	HB	162/146/40	149/172/61	IS	0.38
Ding, 2013 [12]	miR-149-rs71428439	China	Sequencing	NA	95/130/64	132/126/38	CAD	0.42
Buraczynska, 2014 [5]	miR-196a2-rs11614913	Poland	PCR-RFLP	PB	85/240/209	125/417/292	CVD	0.25
Chen, 2014 [7]	miR-196a2-rs11614913	China	PCR-LDR	NA	312/450/157	322/406/161	CAD	0.11
Huang, 2015-a [19]	miR-196a2-rs11614913	China	TaqMan	HB	190/381/147	204/360/156	CAD	0.95
Huang, 2015-b [20]	miR-196a2-rs11614913	China	TaqMan	HB	166/265/100	153/266/112	IS	0.91
Jeon, 2013 [22]	miR-196a2-rs11614913	South Korea	PCR-RFLP	HB	297/533/221	156/292/105	IS	0.14
Liu, 2014 [27]	miR-196a2-rs11614913	China	PCR-RFLP	HB	64/181/51	93/214/84	IS	0.07
Luo, 2017 [29]	miR-196a2-rs11614913	China	SNaPshot	HB	73/138/87	75/159/69	IS	0.43
Park, 2012 [31]	miR-196a2-rs11614913	South Korea	PCR-RFLP	HB	18/64/25	68/115/57	MD	0.60
Sung, 2016 [38]	miR-196a2-rs11614913	South Korea	PCR-RFLP	HB	179/236/107	153/274/108	CAD	0.51
Xiong, 2014 [44]	miR-196a2-rs11614913	China	PCR-RFLP	HB	86/131/78	83/132/68	CAD	0.32
Xu, 2009, I [45]	miR-196a2-rs11614913	China	PCR-RFLP	HB	140/241/120	155/262/88	CHD	0.23
Xu, 2009, II [45]	miR-196a2-rs11614913	China	PCR-RFLP	HB	143/245/114	167/283/91	CHD	0.13
Xu, 2009, III [45]	miR-196a2-rs11614913	China	PCR-RFLP	HB	77/168/76	233/380/124	CHD	0.16
Yang, 2012 [46]	miR-196a2-rs11614913	China	TaqMan	NA	202/463/163	241/463/217	CAD	0.89
Yu, 2016 [48]	miR-196a2-rs11614913	China	TaqMan	PB	93/152/52	75/137/64	CHD	0.98
Zhi, 2012 [50]	miR-196a2-rs11614913	China	PCR-RFLP	PB	291/470/155	208/278/98	CAD	0.80
Zhou, 2010 [52]	miR-196a2-rs11614913	China	PCR-RFLP	HB	93/111/17	86/176/59	DM	0.07
Zhu, 2014 [54]	miR-196a2-rs11614913	China	PCR-LDR	HB	108/189/71	105/198/78	IS	0.43
Zhu, 2017 [53]	miR-196a2-rs11614913	China	TaqMan	HB	150/273/100	146/260/104	IS	0.40
Zhu, 2016 [42]	miR-196a2-rs11614913	China	PCR-RFLP	HB	112/205/79	110/196/72	IS	0.59
Chen, 2014 [7]	miR-499-rs3746444	China	PCR-LDR	NA	612/237/70	606/246/37	CAD	0.08
Chen, 2017 [10]	miR-499-rs3746444	China	MassArray	HB	264/110/47	342/103/19	CAD	0.00
Fawzy, 2018 [13]	miR-499-rs3746444	Egypt	TaqMan	PB	103/116/74	64/42/15	CAD	0.09
Huang, 2015-b [20]	miR-499-rs3746444	China	TaqMan	HB	398/133/0	403/128/0	IS	0.00
Jeon, 2013 [22]	miR-499-rs3746444	South Korea	PCR-RFLP	HB	688/330/33	365/170/18	IS	0.83
Labbaf, 2017	miR-499-rs3746444	Iran	PCR-RFLP	HB	68/142/78	48/77/25	CAD	0.61
Liu, 2014 [27]	miR-499-rs3746444	China	PCR-RFLP	HB	181/96/19	278/99/14	IS	0.23
Luo, 2017 [29]	miR-499-rs3746444	China	SNaPshot	HB	215/78/5	244/53/6	IS	0.22
Lyu, 2016 [14]	miR-499-rs3746444	China	TaqMan	HB	257/110/11	250/113/15	IS	0.72
Park, 2012 [31]	miR-499-rs3746444	South Korea	PCR-RFLP	HB	76/27/4	163/71/6	MD	0.73
Sung, 2016 [38]	miR-499-rs3746444	South Korea	PCR-RFLP	HB	358/155/9	354/168/13	CAD	0.23
Xiong, 2014 [44]	miR-499-rs3746444	China	PCR-RFLP	HB	227/65/3	212/67/4	CAD	0.78
Xu, 2009, I [45]	miR-499-rs3746444	China	PCR-RFLP	HB	373/123/5	367/118/20	CHD	0.02
Xu, 2009, II [45]	miR-499-rs3746444	China	PCR-RFLP	HB	373/113/16	407/121/13	CHD	0.35
Yang, 2012 [46]	miR-499-rs3746444	China	TaqMan	NA	589/210/28	683/212/28	CAD	0.03
Yu, 2016 [48]	miR-499-rs3746444	China	TaqMan	PB	209/82/6	195/76/5	CHD	0.56
Zhi, 2012 [50]	miR-499-rs3746444	China	PCR-RFLP	PB	629/201/86	396/167/21	CAD	0.60
Zhou, 2010 [52]	miR-499-rs3746444	China	PCR-RFLP	HB	104/104/13	219/83/19	DM	0.01
Zhu, 2017 [53]	miR-499-rs3746444	China	TaqMan	HB	349/124/32	328/158/24	IS	0.96
Zhu, 2016 [42]	miR-499-rs3746444	China	PCR-RFLP	HB	255/123/18	249/116/13	IS	0.44

^a^: The source of the control group, either hospital based (HB) or population based (PB); ^b^: The genotype counts in cases or controls, represented as CC/GC/GG (for miR-146a), TT/TC/CC (for miR-149 rs2292832 and miR-196a2 rs11614913) or AA/AG/GG (for miR-149 rs71428439 and miR-499 rs3746444); ^c^: The *p* values for the departure of the control’s genotype frequencies from Hardy-Weinberg equilibrium (HWE). Abbreviations: ACS—acute coronary syndrome; CHD—congenital heart disease; CAD—coronary artery disease; CE—capillary electrophoresis; DC—dilated cardiomyopathy; HB—hospital based; IA—intracranial Aneurism; IS—ischemic stroke; MD—moyamoya disease; MI—myocardial infarction; NA—not available or not mentioned; PB—population based.

**Table 2:** Summary results for meta-analysis of the association between six miRNA polymorphisms and CCD risk.

Genetic Models	*n* ^a^	Samples	OR ^b^ (95% CI)	*P* ^c^	*P*_Het_^d^	I^2^	τ	*P*_bias_^e^
miR-146a rs2910164								
Homozygote (GG vs. CC)	30	13186/14497	0.99 (0.85–1.15)	0.86	<0.01	67.2	0.29	0.94
Heterozygote (GC vs. CC)	30	13186/14497	0.97 (0.90–1.05)	0.44	0.03	35.7	0.12	0.83
Dominant (GG+GC vs. CC)	30	13186/14497	0.98 (0.89–1.07)	0.58	<0.01	57.5	0.17	0.89
Recessive (GG vs. GC+CC)	30	13186/14497	1.00 (0.90–1.13)	0.94	<0.01	58.9	0.21	0.61
Allelic (G vs. C)	30	13186/14497	0.99 (0.92–1.06)	0.72	<0.01	68.7	0.14	0.94
miR-149 rs2292832								
Homozygote (CC vs. TT)	9	4116/3511	1.11 (0.84–1.46)	0.40	0.04	51.1	0.24	-
Heterozygote (CT vs. TT)	9	4116/3511	1.06 (0.96–1.17)	0.23	0.12	38.0	0.12	-
Dominant (CT+CC vs. TT)	9	4116/3511	1.08 (0.99–1.19)	0.10	0.07	45.0	0.13	-
Recessive (CC vs. TT+CT)	9	4116/3511	1.11 (0.97–1.28)	0.13	0.19	29.2	0.14	-
Allelic (C vs. T)	9	4116/3511	1.07 (1.00–1.15)	0.05	0.07	44.9	0.09	-
miR-149 rs71428439								
Homozygote (GG vs. AA)	3	1556/1567	1.21 (0.23–6.36)	0.66	<0.01	87.7	0.54	-
Heterozygote (GA vs. AA)	3	1556/1567	1.04 (0.51–2.12)	0.82	0.043	68.0	0.21	-
Dominant (GA+GG vs. AA)	3	1556/1567	1.09(0.41–2.89)	0.73	<0.01	84.2	0.31	-
Recessive (GG vs. AA+GA)	3	1556/1567	1.18 (0.33–4.17)	0.62	<0.01	82.2	0.40	-
Allelic (G vs. A)	3	1556/1567	1.10 (0.46–2.60)	0.68	<0.01	89.4	0.29	-
miR-196a2 rs11614913								
Homozygote (CC vs. TT)	20	10144/10433	1.02 (0.87–1.20)	0.76	<0.01	60.2	0.23	0.59
Heterozygote (CT vs. TT)	20	10144/10433	1.02 (0.92–1.12)	0.73	0.03	41.2	0.13	0.49
Dominant (CT+CC vs. TT)	20	10144/10433	1.02 (0.92–1.13)	0.70	0.01	49.5	0.14	0.55
Recessive (CC vs. TT+CT)	20	10144/10433	1.01 (0.89–1.16)	0.82	<0.01	60.3	0.19	0.46
Allelic (C vs. T)	20	10144/10433	1.01 (0.94–1.09)	0.70	<0.01	59.6	0.11	0.57
miR-218 rs11134527								
Homozygote (GG vs. AA)	3	2322/2754	0.96 (0.81–1.13)	0.68	0.39	0	0	-
Heterozygote (GA vs. AA)	3	2322/2754	0.95 (0.84–1.08)	0.51	0.43	0	0	-
Dominant (GA+GG vs. AA)	3	2322/2754	0.96 (0.85–1.08)	0.51	0.79	0	0	-
Recessive (GG vs. AA+GA)	3	2322/2754	0.98 (0.85–1.14)	0.86	0.10	54.7	0.15	-
Allelic (G vs. A)	3	2322/2754	0.97 (0.90–1.05)	0.59	0.67	0	0	-
miR-499 rs3746444								
Homozygote (GG vs. AA)	19	9033/8345	1.41 (1.06–1.87)	0.02	<0.01	59.7	0.42	0.81
Heterozygote (GA vs. AA)	20	9564/8876	1.10 (0.95–1.26)	0.18	<0.01	67.7	0.22	0.07
Heterozygote-Trim&fill ^f^	-	-	1.10 (0.95–1.26)	0.18	-	-	-	-
Heterozygote-Copas ^f^	-	-	1.05 (0.94–1.17)	0.35	-	-	-	-
Dominant (GA+GG vs. AA)	20	9564/8876	1.15 (0.99–1.32)	0.05	<0.01	69.0	0.22	0.18
Recessive (GG vs. AA+GA)	19	9033/8345	1.35 (1.03–1.77)	0.03	<0.01	57.7	0.40	0.44
Allelic (G vs. A)	20	9564/8876	1.16 (1.03–1.30)	0.02	<0.01	71.3	0.20	0.91

^a^: The number of studies; ^b^: The pooled OR and 95% CI (Random-effect model); ^c^: The *p* value of the Z-test; ^d^: The *p* value of the Q-test; ^e^: The *p* value of the Harbord (when τ^2^ < 0.1) or the arcsine test (when τ^2^ > 0.1) for funnel plot asymmetry test. f: For these models, “trim and fill” adjusted and Copas adjusted results are shown alongside with the original results.

**Table 3:** Meta-analysis of miR-146a rs2910164 and CCD risk subgrouped by the category and type of disease.

Genetic Models	*n* ^a^	Samples	OR ^b^ (95% CI)	*P* ^c^	*P*_Het_^d^	I^2^	τ	M ^e^
Disease category: CVD								
Homozygote (GG vs. CC)	12	5394/6298	0.84 (0.68–1.05)	0.12	<0.01	62.3	0.25	RE
Homozygote HWE	11	5126/5288	0.79 (0.66–0.94)	<0.01	0.14	32.1	0.13	FE
Homozygote HWD-adj	12	5394/6298	0.84 (0.67–1.06)	0.13	<0.01	64.0	0.26	RE
Heterozygote (GC vs. CC)	12	5394/6298	0.85 (0.78–0.93)	<0.01	0.70	0.0	0.00	FE
Dominant (GG+GC vs. CC)	12	5394/6298	0.85 (0.79–0.93)	<0.01	0.16	29.1	0.10	FE
Recessive (GG vs. GC+CC)	12	5394/6298	0.93 (0.78–1.12)	0.42	<0.01	65.6	0.22	RE
Allelic (G vs. C)	12	5394/6298	0.91 (0.82–1.02)	0.10	<0.01	64.0	0.13	RE
Disease category: CBVD								
Homozygote (GG vs. CC)	17	7041/8570	1.04 (0.76–1.44)	0.78	<0.01	77.7	0.42	RE
Heterozygote (GC vs. CC)	17	7041/8570	1.05 (0.98–1.13)	0.19	0.06	37.5	0.12	FE
Dominant (GG+GC vs. CC)	17	7041/8570	1.05 (0.91–1.21)	0.51	<0.01	67.6	0.21	RE
Recessive (GG vs. GC+CC)	17	7041/8570	1.02 (0.78–1.34)	0.87	<0.01	72.6	0.33	RE
Allelic (G vs. C)	17	7041/8570	1.01 (0.89–1.16)	0.83	<0.01	80.3	0.21	RE
Disease type: CAD								
Homozygote (GG vs. CC)	11	5173/5977	0.82 (0.65–1.03)	0.08	<0.01	63.0	0.25	RE
Homozygote HWE	10	4905/4967	0.78 (0.69–0.88)	<0.01	0.22	24.2	0.11	FE
Homozygote HWD-adj	11	5173/5977	0.82 (0.65–1.04)	0.09	<0.01	64.9	0.26	RE
Heterozygote (GC vs. CC)	11	5173/5977	0.84 (0.76–0.92)	<0.01	0.74	0.0	0.00	FE
Dominant (GG+GC vs. CC)	11	5173/5977	0.84 (0.77–0.92)	<0.01	0.19	26.2	0.09	FE
Recessive (GG vs. GC+CC)	11	5173/5977	0.92 (0.75–1.11)	0.34	<0.01	67.5	0.22	RE
Allelic (G vs. C)	11	5173/5977	0.90 (0.80–1.01)	0.07	<0.01	64.7	0.13	RE
Disease type: IS								
Homozygote (GG vs. CC)	13	5628/7175	1.09 (0.72–1.65)	0.66	<0.01	81.3	0.45	RE
Heterozygote (GC vs. CC)	13	5628/7175	1.04 (0.91–1.18)	0.57	0.03	48.3	0.14	FE
Dominant (GG+GC vs. CC)	13	5628/7175	1.04 (0.86–1.25)	0.66	<0.01	74.3	0.24	RE
Recessive (GG vs. GC+CC)	13	5628/7175	1.08 (0.78–1.52)	0.61	<0.01	75.5	0.34	FE
Allelic (G vs. C)	13	5628/7175	1.02 (0.86–1.22)	0.77	<0.01	84.3	0.23	RE

^a^: The number of studies; ^b^: The pooled OR and 95% CI (Random-effects model); ^c^: The *p* value of the Z-test; ^d^: The *p* value of the Q-test; ^e^: Either the random-effects (RE) or fixed-effects (FE) model. Abbreviations: CVD—cardiovascular diseases; CBVD—cerebrovascular diseases; CAD—coronary artery disease; IS—ischemic stroke.

**Table 4:** Meta-analysis of miR-149 rs2292832 and CCD risk in the CBVD and the IS subgroups.

Genetic Models	*n* ^a^	Samples	OR ^b^ (95% CI)	*P* ^c^	*P*_Het_^d^	I^2^	τ	M ^e^
Disease category: CBVD (IS, SBI)								
Homozygote (CC vs. TT)	6	2821/2322	1.25 (1.04–1.50)	0.02	0.09	47.9	0.22	FE
Heterozygote (CT vs. TT)	6	2821/2322	1.06 (0.95–1.20)	0.30	0.53	0.0	0.00	FE
Dominant (CT+CC vs. TT)	6	2821/2322	1.10 (0.99–1.24)	0.08	0.27	21.6	0.07	FE
Recessive (CC vs. TT+CT)	6	2821/2322	1.22 (1.03–1.45)	0.02	0.17	35.5	0.16	FE
Allelic (C vs. T)	6	2821/2322	1.11 (1.02–1.20)	0.02	0.10	46.3	0.10	FE
Disease type: IS								
Homozygote (CC vs. TT)	6	2448/2322	1.31 (1.09–1.58)	<0.01	0.14	39.6	0.19	FE
Heterozygote (CT vs. TT)	6	2448/2322	1.07 (0.95–1.21)	0.26	0.52	0.0	0.00	FE
Dominant (CT+CC vs. TT)	6	2448/2322	1.12 (1.00–1.26)	0.047	0.28	20.7	0.07	FE
Recessive (CC vs. TT+CT)	6	2448/2322	1.28 (1.08–1.52)	0.01	0.29	18.6	0.10	FE
Allelic (C vs. T)	6	2448/2322	1.13 (1.04–1.23)	<0.01	0.13	41.1	0.09	FE

^a^: The number of studies; ^b^: The pooled OR and 95% CI; ^c^: The *p* value of the Z-test; ^d^: The *p* value of the Q-test; ^e^: Either the random-effects (RE) or fixed-effects (FE) model. Abbreviations: CBVD—cerebrovascular diseases; IS—ischemic stroke; SBI—silent brain infarction.

**Table 5:** Meta-analysis of miR-196a2 rs11614913 and CCD risk subgrouped by the category and type of disease.

Genetic Models	*n* ^a^	Samples	OR ^b^ (95% CI)	*P* ^c^	*P*_Het_^d^	I^2^	τ	M ^e^
Disease category: CHD								
Homozygote (CC vs. TT)	4	1621/2059	1.31 (0.65–2.64)	0.31	0.01	75.1	0.34	RE
Heterozygote (CT vs. TT)	4	1621/2059	1.06 (0.91–1.24)	0.43	0.39	1.1	0.02	FE
Dominant (CT+CC vs. TT)	4	1621/2059	1.14 (0.99–1.32)	0.07	0.11	49.5	0.15	FE
Recessive (CC vs. TT+CT)	4	1621/2059	1.26 (0.71–2.25)	0.28	0.01	72.5	0.28	RE
Allelic (C vs. T)	4	1621/2059	1.13 (0.82–1.57)	0.32	0.01	72.8	0.16	RE
Disease category: CVD								
Homozygote (CC vs. TT)	7	4419/4253	0.89 (0.61–1.29)	0.47	<0.01	68.5	0.25	RE
Heterozygote (CT vs. TT)	7	4419/4253	0.99 (0.77–1.27)	0.94	<0.01	69.2	0.20	RE
Dominant (CT+CC vs. TT)	7	4419/4253	0.96 (0.74–1.25)	0.71	<0.01	73.1	0.21	RE
Recessive (CC vs. TT+CT)	7	4419/4253	0.89 (0.69–1.15)	0.33	0.04	55.3	0.16	RE
Allelic (C vs. T)	7	4419/4253	0.95 (0.79–1.13)	0.48	<0.01	71.1	0.13	RE
Disease category: CBVD								
Homozygote(CC vs. TT)	8	3570/3287	1.01 (0.88–1.16)	0.90	0.57	0.0	0.00	FE
Heterozygote (CT vs. TT)	8	3570/3287	1.01 (0.90–1.13)	0.82	0.33	12.7	0.06	FE
Dominant(CT+CC vs. TT)	8	3570/3287	1.01 (0.91–1.13)	0.83	0.46	0.0	0.00	FE
Recessive(CC vs. TT+CT)	8	3570/3287	1.00 (0.89–1.13)	0.99	0.38	6.0	0.04	FE
Allelic (C vs. T)	8	3570/3287	1.00 (0.94–1.08)	0.89	0.62	0.0	0.00	FE
Disease type: CAD								
Homozygote(CC vs. TT)	6	4198/3932	0.99 (0.87–1.12)	0.84	0.81	0.0	0.00	FE
Heterozygote (CT vs. TT)	6	4198/3932	1.08 (0.98–1.20)	0.11	0.08	49.0	0.13	FE
Dominant (CT+CC vs. TT)	6	4198/3932	1.06 (0.96–1.16)	0.25	0.19	32.4	0.08	FE
Recessive (CC vs. TT+CT)	6	4198/3932	0.94 (0.84–1.04)	0.24	0.61	0.0	0.00	FE
Allelic (C vs. T)	6	4198/3932	1.00 (0.94–1.07)	0.93	0.63	0.0	0.00	FE
Disease type: IS								
Homozygote (CC vs. TT)	7	3090/3047	0.98 (0.85–1.14)	0.82	0.73	0.0	0.00	FE
Heterozygote (CT vs. TT)	7	3090/3047	0.99 (0.88–1.12)	0.92	0.91	0.0	0.00	FE
Dominant (CT+CC vs. TT)	7	3090/3047	0.99 (0.89–1.11)	0.89	0.95	0.0	0.00	FE
Recessive (CC vs. TT+CT)	7	3090/3047	0.99 (0.87–1.12)	0.87	0.33	13.3	0.07	FE
Allelic (C vs. T)	7	3090/3047	0.99 (0.93–1.07)	0.85	0.75	0.0	0.00	FE

^a^: The number of studies; ^b^: The pooled OR and 95% CI (Random-effect model); ^c^: The *p* value of the Z-test; ^d^: The *p* value of the Q-test; ^e^: Either the random-effects (RE) or fixed-effects (FE) model. Abbreviations: CHD—congenital heart disease; CVD—cardiovascular disease; CBVD—cerebrovascular disease; CAD—coronary artery disease; IS—ischemic stroke.

**Table 6:** Meta-analysis of miR-499 rs3746444 and CCD risk subgrouped by the category and type of disease.

Genetic Models	*n* ^a^	Samples	OR ^b^ (95% CI)	*P* ^c^	*P*_Het_^d^	I^2^	τ	M ^e^
Disease category: CHD								
Homozygote (GG vs. AA)	3	1300/1322	0.73 (0.07–7.44)	0.61	0.02	74.0	0.83	RE
Heterozygote	3	1300/1322	1.02 (0.85–1.22)	0.84	1.00	0.0	0.00	FE
Dominant	3	1300/1322	0.99 (0.83–1.17)	0.88	0.77	0.0	0.00	FE
Recessive	3	1300/1322	0.72 (0.07–7.43)	0.60	0.02	74.3	0.83	RE
Allelic (G vs. A)	3	1300/1322	0.96 (0.82–1.12)	0.59	0.31	14.3	0.06	FE
Disease category: CVD								
Homozygote (GG vs. AA)	9	4702/4270	1.84 (1.24–2.73)	<0.01	0.02	55.9	0.35	RE
Heterozygote	9	4702/4270	1.18 (0.88–1.58)	0.22	0.00	80.6	0.31	RE
Dominant	9	4702/4270	1.29 (0.97–1.70)	0.07	0.00	79.9	0.28	RE
Recessive	9	4702/4270	1.70 (1.15–2.50)	0.01	0.02	57.2	0.34	RE
Allelic (G vs. A)	9	4702/4270	1.30 (1.04–1.62)	0.02	0.00	78.4	0.22	RE
Disease category: CBVD								
Homozygote (GG vs. AA)	8	3562/3284	1.19 (0.90–1.58)	0.21	0.57	0.0	0.00	FE
Heterozygote	8	3562/3284	1.05 (0.85–1.31)	0.59	0.02	58.6	0.19	RE
Dominant	8	3562/3284	1.07 (0.87–1.31)	0.45	0.03	56.0	0.17	RE
Recessive	8	3562/3284	1.20 (0.91–1.57)	0.20	0.64	0.0	0.00	FE
Allelic (G vs. A)	8	3562/3284	1.06 (0.97–1.16)	0.20	0.06	48.6	0.13	FE
Disease type: CAD								
Homozygote (GG vs. AA)	8	4405/3949	1.91 (1.19–3.07)	0.01	0.01	62.8	0.39	RE
Heterozygote	8	4405/3949	1.06 (0.85–1.34)	0.54	0.01	63.6	0.19	RE
Dominant	8	4405/3949	1.20 (0.92–1.57)	0.16	0.00	72.8	0.23	RE
Recessive	8	4405/3949	1.82 (1.19–2.77)	0.01	0.02	57.3	0.34	RE
Allelic (G vs. A)	8	4405/3949	1.26 (0.99–1.62)	0.06	0.00	79.0	0.22	RE
Disease type: IS								
Homozygote (GG vs. AA)	7	3082/3044	1.20 (0.90–1.60)	0.21	0.48	0.0	0.00	FE
Heterozygote	7	3082/3044	1.06 (0.82–1.36)	0.61	0.01	64.9	0.21	RE
Dominant	7	3082/3044	1.07 (0.85–1.36)	0.49	0.01	63.5	0.20	RE
Recessive	7	3082/3044	1.21 (0.91–1.61)	0.19	0.58	0.0	0.00	FE
Allelic (G vs. A)	7	3082/3044	1.08 (0.89–1.30)	0.38	0.03	58.0	0.15	RE

^a^: Samples are shown as number of cases/number of controls; ^b^: The pooled OR and 95% CI (Random-effect model); ^c^: The *p* value of the Z-test; ^d^: The *p* value of the Q-test; ^e^: Either the random-effects (RE) or fixed-effects (FE) model. Abbreviations: CHD—congenital heart disease; CVD—cardiovascular disease; CBVD—cerebrovascular disease; CAD—coronary artery disease; IS—ischemic stroke.

## Supplementary Materials

Supplementary materials can be found at http://www.mdpi.com/1422-0067/20/2/293/s1.

## Abbreviations

CCD	Cardiocerebrovascular diseases
CVD	Cardiovascular diseases
CBVD	Cerebrovascular diseases
CHD	Congenital heart disease
CAD	Coronary artery disease
CI	Confidence interval
FE	Fixed-effects model
HWE	Hardy-Weinberg equilibrium
HWD	Deviation from HWE
IS	Ischemic stroke
OR	Odds ratio
RE	Random-effects model
SBI	Silent brain infarction
SNP	Single nucleotide polymorphism
