# Supplementary material for: miRNA Polymorphisms and Risk of Cardio-Cerebrovascular Diseases: A Systematic Review and Meta-Analysis
Source: Int J Mol Sci. 2019 Jan 12;20(2):293. doi: 10.3390/ijms20020293 (PMC6359604; doi:10.3390/ijms20020293)
Supplement: Supplementary file 1 [file ijms-20-00293-s001.zip › Table S1.docx]

**Supplementary Table S1.** **Characteristics of the identified studies on the association between miRNA polymorphisms and CCD risk which were not included in the meta-analysis.** A total of 25 miRNA polymorphisms were evaluated in these studies. The number of studies per each polymorphism was less than three, and, therefore, these studies were not included in meta-analysis.

| **Author** | **Country** | **Genotyping Method** | **Source** | **miRNA polymorphism A>B^a^** | **Cases^b^** | **Controls** | **Disease** | **HWE^c^** |
| --- | --- | --- | --- | --- | --- | --- | --- | --- |
|  |  |  |  |  | **AA/AB/BB** | **AA/AB/BB** |  |  |
| Zhang, 2016 | China | PCR-RFLP | PB | Let-7-rs10877887 T>C | 164/131/34 | 168/157/32 | IS | 1 |
| Sima, 2015 | China | PCR-RFLP | HB | Let-7-rs10877887 T>C | 157/117/31 | 192/167/42 | IA | 0 |
| Zhang, 2016 | China | TaqMan | PB | Let-7-rs13293512T>C | 134/158/37 | 116/167/74 | IS | 1 |
| Sima, 2015 | China | TaqMan | HB | Let-7-rs13293512 T>C | 71/177/57 | 122/209/70 | IA | 1 |
| Cai, 2018 | China | PCR-LDR | HB | miR-100-rs1834306C>T | 99/143/45 | 221/300/125 | MI | 0 |
| Xiang, 2017 | China | Snapshot | PB | miR-126-rs4636297G>A | 430/147/15 | 275/154/27 | IS | 1 |
| Cai, 2018 | China | PCR-LDR | HB | miR-126-rs4636297G>A | 216/65/6 | 471/164/11 | MI | 1 |
| Choi, 2016 | South Korea | PCR-RFLP | HB | miR‑130a-rs731384C>T | 479/110/7 | 328/74/2 | IS | 1 |
| Kim, 2016 | South Korea | TaqMan | PB | miR-130b-rs373001T>C | 290/192/41 | 228/144/28 | IS | 1 |
| Gao, 2018 | China | Sequencing | HB | miR-138-rs139365823C>A | 833/24/0 | 925/13/0 | CHD | 0 |
| Gao, 2018 | China | Sequencing | HB | miR-138-rs76987351G>A | 717/135/5 | 761/169/8 | CHD | 1 |
| Wei, 2016 | China | TaqMan | HB | miR-143/145-rs4705342T>C | 234/174/37 | 228/232/58 | IS | 1 |
| Wei, 2016 | China | PCR-RFLP | HB | miR-143/145-rs4705343T>C | 188/209/48 | 230/243/45 | IS | 1 |
| Yang, 2014 | China | TaqMan | HB | miR-143-rs41291957G>A | 484/487/138 | 414/387/114 | CHD | 1 |
| Wang, 2017 | China | MassARRAY | HB | miR-146a-rs2431697C>T | 219/111/23 | 254/102/12 | CAD | 1 |
| Zhong, 2016 | China | CE | HB | miR-146a-rs57095329A>G | 200/87/10 | 211/82/7 | IS | 1 |
| Choi, 2016 | South Korea | PCR-RFLP | HB | miR-150-rs73056059G>A | 544/52/0 | 380/24/0 | IS | 1 |
| Choi, 2016 | South Korea | PCR-RFLP | HB | miR-155-rs767649T>A | 167/311/118 | 117/191/96 | IS | 1 |
| Kim, 2016 | South Korea | PCR-RFLP | PB | miR-200b-rs7549819T>C | 238/236/49 | 174/177/49 | IS | 1 |
| Xiang, 2017 | China | Snapshot | PB | miR-21-rs1292037T>C | 172/304/116 | 152/229/75 | IS | 1 |
| Cai, 2018 | China | PCR-LDR | HB | miR-26a-1-rs7372209C>T | 162/109/16 | 370/240/36 | MI | 0 |
| Yu, 2016 | China | TaqMan | PB | miR-27a-rs895819A>G | 165/115/17 | 150/115/11 | CHD | 1 |
| Cai, 2018 | China | PCR-LDR | HB | miR-27a-rs895819 A>G | 156/102/29 | 358/251/37 | MI | 1 |
| Choi, 2016 | South Korea | PCR-RFLP | HB | miR-34a-rs6577555C>A | 323/229/44 | 239/143/22 | IS | 1 |
| Liu, 2013 | China | TaqMan | HB | miR-34b/c-rs4938723T>C | 258/265/67 | 306/296/70 | CHD | 1 |
| Kim, 2016 | South Korea | PCR-RFLP | PB | miR-495-rs2281611A>C | 123/280/120 | 102/196/102 | IS | 1 |
| Xiang, 2017 | China | Snapshot | PB | miR-605-rs2043556T>C | 332/232/28 | 276/153/27 | IS | 1 |

**a:** A and B represent alleles of each polymorphism; **b:** The A and B allele for each polymorphism are given in the previous column. **c**: 1 indicate that the genotype counts in control group was in agreement with HWE, while zero indicates a statistically significant departure from HWE.

**Abbreviations:** ACS: acute coronary syndrome; CE: Capillary Electrophoresis; CHD: congenital heart disease; CAD: coronary artery disease; HB: Hospital-based; IA: intracranial Aneurism; IS: ischemic stroke; MI: myocardial infarction; PB: Population-based;
