# Supplementary material for: miRNA Polymorphisms and Risk of Cardio-Cerebrovascular Diseases: A Systematic Review and Meta-Analysis
Source: Int J Mol Sci. 2019 Jan 12;20(2):293. doi: 10.3390/ijms20020293 (PMC6359604; doi:10.3390/ijms20020293)
Supplement: Supplementary file 1 [file ijms-20-00293-s001.zip › Table S2.docx]

**Supplementary Table S2.** HWE sensitivity analysis for meta-analysis of miR-146a rs2910164 and CCD risk in genotypic contrasts. Only studies with the control groups in HWE were included in meta-analysis. The results of overall and subgroup analysis are consistent with the results of original meta-analysis (compare with Table 2 and Table 3), except for the homozygote comparison of the CVD and the CAD subgroups in which results were adjusted for HWD.

| **Genetic Models** | **n^a^** | **Samples** | **OR^b^ (95% CI)** | ***P*^c^** | ***P*_Het_^d^** | **I*^2^*** | **τ** | **M^e^** |
| --- | --- | --- | --- | --- | --- | --- | --- | --- |
| **Overall analysis** | | | | | | | | |
| Homozygote(GG vs. CC) | 28 | 11632/11902 | 0.99 (0.84-1.17) | 0.91 | <0.01 | 69.3 | 0.32 | RE |
| Heterozygote(GC vs. CC) | 28 | 11632/11902 | 0.97 (0.90-1.06) | 0.52 | 0.02 | 40.1 | 0.13 | RE |
| Dominant(GG+GC vs. CC) | 28 | 11632/11902 | 0.98 (0.89-1.08) | 0.65 | <0.01 | 60.3 | 0.19 | RE |
| Recessive(GG vs. GC+CC) | 28 | 11632/11902 | 1.01 (0.89-1.14) | 0.89 | <0.01 | 61.6 | 0.23 | RE |
| ***Disease category: CVD*** | | | | | | | | |
| Homozygote(GG vs. CC) | 11 | 5126/5288 | 0.80 (0.71-0.89) | **<0.01** | 0.14 | 32.2 | 0.14 | FE |
| Heterozygote(GC vs. CC) | 11 | 5126/5288 | 0.83 (0.76-0.92) | **<0.01** | 0.79 | 0.0 | 0.00 | FE |
| Dominant(GG+GC vs. CC) | 11 | 5126/5288 | 0.83 (0.76-0.90) | **<0.01** | 0.56 | 0.0 | 0.00 | FE |
| Recessive(GG vs. GC+CC) | 11 | 5126/5288 | 0.88 (0.75-1.03) | 0.11 | 0.04 | 46.5 | 0.15 | RE |
| ***Disease category: CBVD*** | | | | | | | | |
| Homozygote(GG vs. CC) | 15 | 5755/5975 | 1.14 (0.89-1.47) | 0.27 | <0.01 | 70.2 | 0.36 | RE |
| Heterozygote(GC vs. CC) | 15 | 5755/5975 | 1.08 (0.99-1.17) | 0.05 | 0.08 | 36.3 | 0.12 | FE |
| Dominant(GG+GC vs. CC) | 15 | 5755/5975 | 1.09 (0.95-1.26) | 0.18 | <0.01 | 60.0 | 0.19 | RE |
| Recessive(GG vs. GC+CC) | 15 | 5755/5975 | 1.10 (0.89-1.34) | 0.35 | <0.01 | 61.6 | 0.27 | RE |
| ***Disease type: CAD*** | | | | | | | | |
| Homozygote(GG vs. CC) | 10 | 4905/4967 | 0.78 (0.69-0.88) | **<0.01** | 0.22 | 24.2 | 0.11 | FE |
| Heterozygote(GC vs. CC) | 10 | 4905/4967 | 0.82 (0.75-0.91) | **<0.01** | 0.86 | 0.0 | 0.00 | FE |
| Dominant(GG+GC vs. CC) | 10 | 4905/4967 | 0.81 (0.74-0.89) | **<0.01** | 0.73 | 0.0 | 0.00 | FE |
| Recessive(GG vs. GC+CC) | 10 | 4905/4967 | 0.87 (0.74-1.02) | 0.08 | 0.05 | 46.9 | 0.14 | RE |
| ***Disease type: IS*** | | | | | | | | |
| Homozygote(GG vs. CC) | 11 | 4342/4580 | 1.24 (0.92-1.67) | 0.14 | <0.01 | 73.4 | 0.37 | RE |
| Heterozygote(GC vs. CC) | 11 | 4342/4580 | 1.07 (0.92-1.24) | 0.37 | 0.03 | 50.1 | 0.16 | RE |
| Dominant(GG+GC vs. CC) | 11 | 4342/4580 | 1.10 (0.92-1.32) | 0.27 | <0.01 | 69.0 | 0.22 | RE |
| Recessive(GG vs. GC+CC) | 11 | 4342/4580 | 1.19 (0.96-1.49) | 0.10 | <0.01 | 60.3 | 0.25 | RE |

Samples are shown as number of cases/number of controls; **a:** number of studies; **b**: Pooled OR and 95% CI; **c:** *P-value* of the Z-test; **d**: *P-value* of the Q-test; **e:** either random-effects (RE) or fixed-effects (FE) model. **Abbreviations:** CVD: cardiovascular disease; CBVD: cerebrovascular disease; CAD: coronary artery disease; IS: ischemic stroke.
