# Supplementary material for: miRNA Polymorphisms and Risk of Cardio-Cerebrovascular Diseases: A Systematic Review and Meta-Analysis
Source: Int J Mol Sci. 2019 Jan 12;20(2):293. doi: 10.3390/ijms20020293 (PMC6359604; doi:10.3390/ijms20020293)
Supplement: Supplementary file 1 [file ijms-20-00293-s001.zip › Table S3.docx]

**Supplementary Table S3.** HWE sensitivity analysis for meta-analysis of miR-499 rs3746444 and CCD risk in genotypic contrasts. Only studies with the control groups in HWE were included in meta-analysis. The results of overall and subgroup analysis are consistent with the results of original meta-analysis (compare with Table 2 and Table 6).

| **Genetic Models** | **n^a^** | **Samples** | **OR^b^ (95% CI)** | ***P*^c^** | ***P*_Het_^d^** | **I*^2^*** | **τ** | **M^e^** |
| --- | --- | --- | --- | --- | --- | --- | --- | --- |
| **Overall analysis** | | | | | | | | |
| Homozygote(GG vs. AA) | 15 | 7213/6277 | 1.61 (1.35-1.91) | **<0.01** | 0.05 | 40.9 | 0.29 | FE |
| Heterozygote (GA vs. AA) | 15 |  | 1.02 (0.89-1.17) | 0.77 | 0.01 | 52.6 | 0.17 | RE |
| Dominant (GA+GG vs. AA) | 15 |  | 1.08 (0.94-1.24) | 0.24 | 0.01 | 55.0 | 0.17 | RE |
| Recessive(GG vs. AA+GA) | 15 |  | 1.59 (1.35-1.88) | **<0.01** | 0.09 | 34.2 | 0.25 | FE |
| ***Disease category: CHD*** | | | | | | | | |
| Homozygote(GG vs. AA) | 2 | 799/817 | 1.28 (0.68-2.41) | 0.45 | - | - | - | FE |
| Heterozygote (GA vs. AA) | 2 | 799/817 | 1.01 (0.81-1.28) | 0.90 | - | - | - | FE |
| Dominant (GA+GG vs. AA) | 2 | 799/817 | 1.04 (0.83-1.29) | 0.75 | - | - | - | FE |
| Recessive(GG vs. AA+GA) | 2 | 799/817 | 1.27 (0.68-2.39) | 0.45 | - | - | - | FE |
| Allelic (G vs. A) | 2 | 799/817 | 1.05 (0.87-1.28) | 0.61 | - | - | - | FE |
| ***Disease category: CVD*** | | | | | | | | |
| Homozygote(GG vs. AA) | 6 | 3233/2562 | 1.89 (1.06-3.34) | **0.04** | 0.06 | 53.5 | 0.34 | RE |
| Heterozygote (GA vs. AA) | 6 | 3233/2562 | 0.99 (0.75-1.31) | 0.91 | 0.04 | 56.0 | 0.17 | RE |
| Dominant (GA+GG vs. AA) | 6 | 3233/2562 | 1.12 (0.81-1.55) | 0.41 | 0.01 | 66.9 | 0.21 | RE |
| Recessive(GG vs. AA+GA) | 6 | 3233/2562 | 1.96 (1.56-2.46) | **<0.01** | 0.09 | 48.0 | 0.29 | FE |
| ***Disease category: CBVD*** | | | | | | | | |
| Homozygote(GG vs. AA) | 7 | 3562/3284 | 1.19 (0.90-1.58) | 0.21 | 0.57 | 0.0 | 0.00 | FE |
| Heterozygote (GA vs. AA) | 7 | 3562/3284 | 1.06 (0.81-1.38) | 0.63 | 0.01 | 64.5 | 0.22 | RE |
| Dominant (GA+GG vs. AA) | 7 | 3562/3284 | 1.08 (0.84-1.38) | 0.45 | 0.01 | 62.3 | 0.20 | RE |
| Recessive(GG vs. AA+GA) | 7 | 3562/3284 | 1.20 (0.91-1.57) | 0.20 | 0.64 | 0.0 | 0.00 | FE |
| ***Disease type: CAD*** | | | | | | | | |
| Homozygote(GG vs. AA) | 6 | 3157/2562 | 1.91 (1.03-3.55) | **0.04** | 0.03 | 58.4 | 0.37 | RE |
| Heterozygote (GA vs. AA) | 6 | 3157/2562 | 1.00 (0.73-1.37) | 1.00 | 0.02 | 61.2 | 0.20 | RE |
| Dominant (GA+GG vs. AA) | 6 | 3157/2562 | 1.14 (0.79-1.65) | 0.40 | 0.00 | 71.5 | 0.23 | RE |
| Recessive(GG vs. AA+GA) | 6 | 3157/2562 | 1.84 (1.09-3.12) | **0.03** | 0.07 | 50.5 | 0.30 | RE |
| ***Disease type: IS*** | | | | | | | | |
| Homozygote(GG vs. AA) | 6 | 2551/2513 | 1.20 (0.90-1.60) | 0.21 | 0.48 | 0.0 | 0.00 | FE |
| Heterozygote (GA vs. AA) | 6 | 2551/2513 | 1.06 (0.77-1.46) | 0.65 | 0.00 | 70.7 | 0.24 | RE |
| Dominant (GA+GG vs. AA) | 6 | 2551/2513 | 1.08 (0.80-1.46) | 0.53 | 0.01 | 69.6 | 0.23 | RE |
| Recessive(GG vs. AA+GA) | 6 | 2551/2513 | 1.21 (0.91-1.61) | 0.19 | 0.58 | 0.0 | 0.00 | FE |

Samples are shown as number of cases/number of controls; **a:** number of studies; **b**: Pooled OR and 95% CI (Random-effect model); **c:** *Pvalue* of the Z-test; **d**: *Pvalue* of the Q-test; **e:** either random-effects (RE) or fixed-effects (FE) model. **Abbreviations:** CHD: congenital heart disease; CVD: cardiovascular disease; CBVD: cerebrovascular disease; CAD: coronary artery disease; IS: ischemic stroke.
