# Supplementary figures and images for: miRNA Polymorphisms and Risk of Cardio-Cerebrovascular Diseases: A Systematic Review and Meta-Analysis
Source: Int J Mol Sci. 2019 Jan 12;20(2):293. doi: 10.3390/ijms20020293 (PMC6359604; doi:10.3390/ijms20020293)

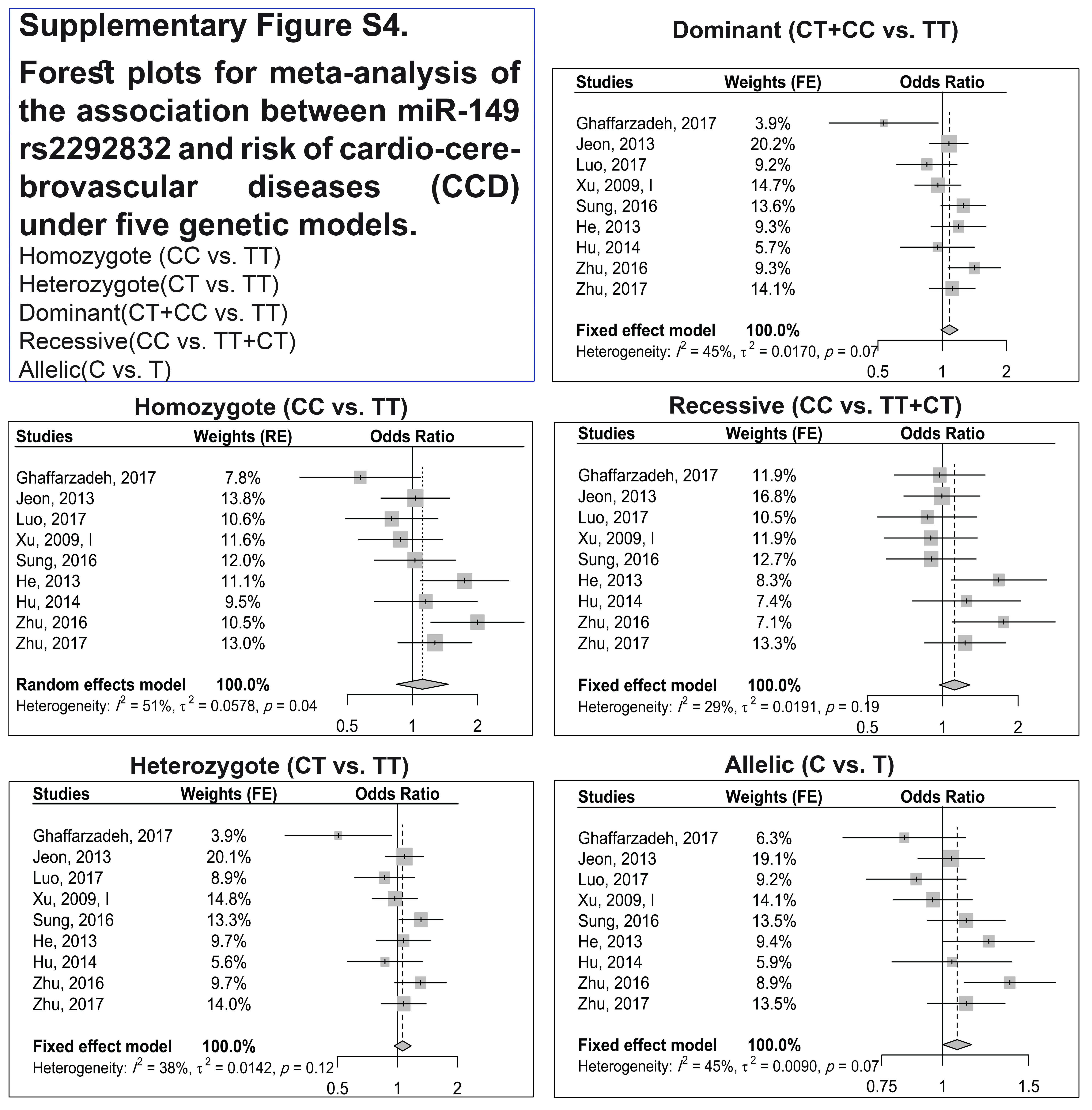

Supplement: Supplementary file 1 [file ijms-20-00293-s001.zip › Figure S4.tif]

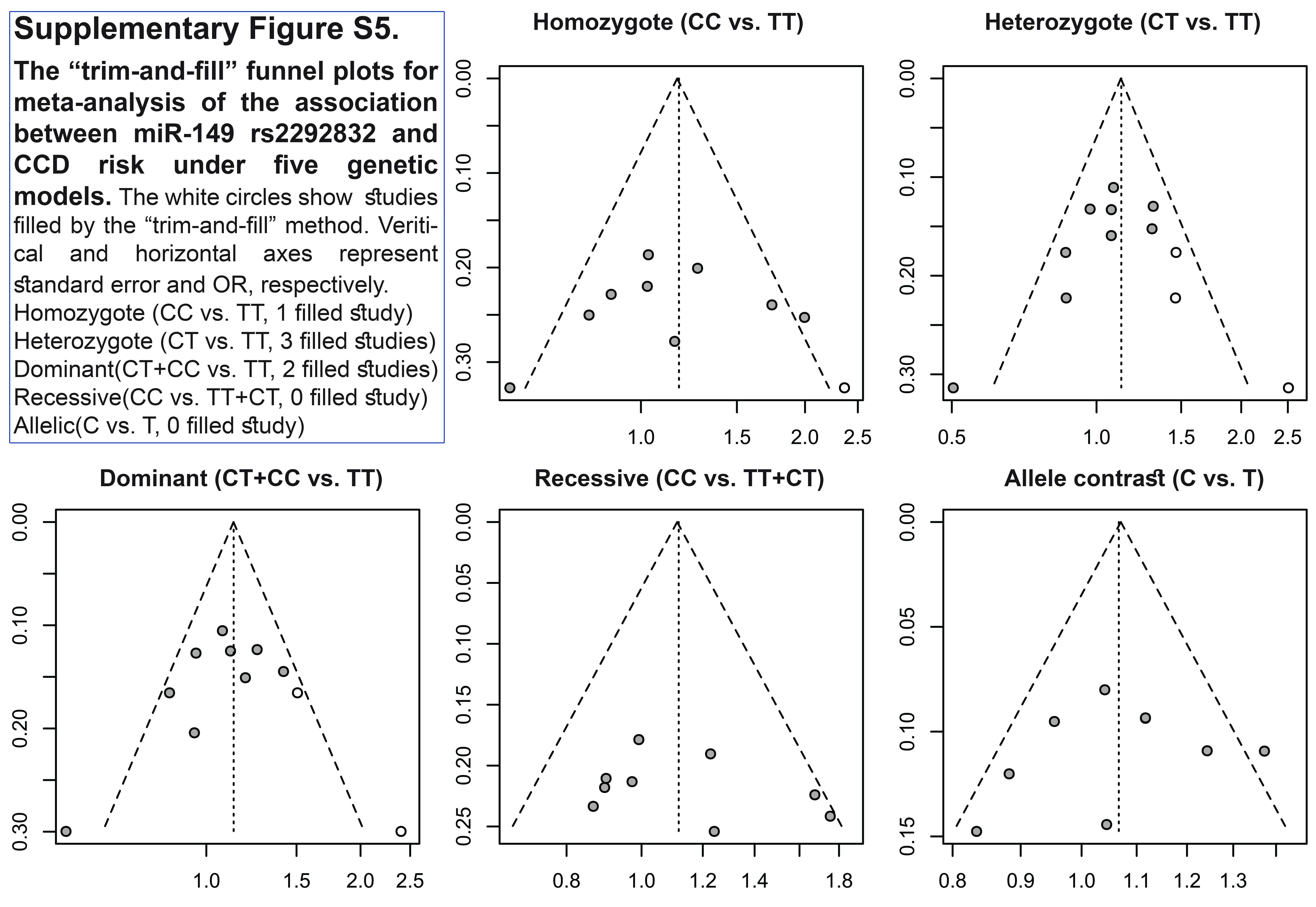

Supplement: Supplementary file 1 [file ijms-20-00293-s001.zip › Figure S5.tif]
